# Unveiling six novel bacterial strains for fipronil and thiobencarb biodegradation: efficacy, metabolic pathways, and bioaugmentation potential in paddy soil

**DOI:** 10.3389/fmicb.2024.1462912

**Published:** 2024-10-22

**Authors:** Nastaran Faridy, Ehssan Torabi, Ahmad Ali Pourbabaee, Ebrahim Osdaghi, Khalil Talebi

**Affiliations:** ^1^Department of Plant Protection, Faculty of Agriculture, University College of Agriculture and Natural Resources, University of Tehran, Karaj, Iran; ^2^Department of Soil Science, Faculty of Agriculture, University College of Agriculture and Natural Resources, University of Tehran, Karaj, Iran

**Keywords:** pesticide, bacteria, transformation products, response surface methodology, degradation rate, bioremediation

## Abstract

**Introduction:**

Soil bacteria offer a promising approach to bioremediate pesticide contamination in agricultural ecosystems. This study investigated the potential of bacteria isolated from rice paddy soil for bioremediating fipronil and thiobencarb, common agricultural pesticides.

**Methods:**

Bacterial isolates capable of degrading fipronil and thiobencarb were enriched in a mineral salt medium. A response surface methodology with a Box-Behnken design was utilized to optimize pesticide degradation with the isolated bacteria. Bioaugmentation tests were performed in paddy soils with varying conditions.

**Results and discussion:**

Six strains, including single isolates and their mixture, efficiently degraded these pesticides at high concentrations (up to 800 µg/mL). *Enterobacter* sp., *Brucella* sp. (alone and combined), and a mixture of *Stenotrophomonas* sp., *Bordetella* sp., and *Citrobacter* sp. effectively degraded fipronil and thiobencarb, respectively. Notably, a single *Pseudomonas* sp. strain degraded a mixture of both pesticides. Optimal degradation conditions were identified as a slightly acidic pH (6-7), moderate pesticide concentrations (20-50 µg/mL), and a specific inoculum size. Bioaugmentation assays in real-world paddy soils (sterile/non-sterile, varying moisture) demonstrated that these bacteria significantly increased degradation rates (up to 14.15-fold for fipronil and 5.13-fold for thiobencarb). The study identifies these novel bacterial strains as promising tools for bioremediation and bioaugmentation strategies to tackle fipronil and thiobencarb contamination in paddy ecosystems.

## 1 Introduction

The widespread application of pesticides within agroecosystems has become a concern due to their potential to cause harm to both human health and the environment (Topping et al., 2020). Fipronil and thiobencarb are two commonly used pesticides in rice paddies, targeting insect pests and unwanted vegetation, respectively (Moinoddini et al., 2014).

Fipronil, classified as a phenylpyrazole insecticide, exhibits moderate to high persistence in soil environments (El-Aswad et al., 2024). Residual fipronil has been documented in cultivated rice field soil, adjacent sediments, and even nearby rivers, highlighting its potential for environmental dispersion (Bhatt et al., 2021a). Additionally, its bioconcentration factor suggests accumulation, posing a potential threat to ecosystem health (Jackson et al., 2009; Qu et al., 2014).

Thiobencarb is a systemic herbicide of the thiocarbamate class. Due to its moderate soil mobility, it presents a risk of contaminating both soil and groundwater resources (Saka, 2010; Wang et al., 2019). The presence of thiobencarb has been confirmed in water samples collected from rice paddies, further supporting concerns regarding its environmental impact (Sapari and Ismail, 2012).

Bioremediation, a process that utilizes soil microorganisms to degrade contaminants, offers a promising strategy for reducing environmental pesticide residues and achieving their complete mineralization (Torabi et al., 2017; Pourbabaee et al., 2018a, 2018b; Pourbabaei et al., 2020). Research has identified various indigenous microbial strains capable of degrading fipronil, including species belonging to the genera *Streptomyces* sp., *Bacillus* sp., *Paracoccus* sp., *Stenotrophomonas* sp., *Klebsiella* sp., *Staphylococcus* sp., *Bacillus thuringiensis*, and *Aspergillus* sp. (Kumar et al., 2012; Uniyal et al., 2016; Gajendiran and Abraham, 2017; Abraham and Gajendiran, 2019; Sayi et al., 2020; Bhatt et al., 2021a, 2021b). Similarly, studies have reported aerobic and anaerobic microbial strains with the ability to degrade thiobencarb, including *Aspergillus niger*, *Corynebacterium* sp., *Acidovorax* sp., *Pseudomonas* sp., *Cupriavidus oxalaticus*, *Dechloromonas* sp., *Thauera* sp., and *Azoarcus* sp. (Miwa et al., 1988; Torra-Reventós et al., 2004; Chu et al., 2017; Duc et al., 2023; Duc, 2023).

Despite existing research on microbial fipronil and thiobencarb degradation, a comprehensive investigation into the bioaugmentation potential of these isolates under diverse soil conditions remains limited. Furthermore, no prior studies have documented the existence of microorganisms capable of simultaneously degrading fipronil and thiobencarb.

This study addresses these knowledge gaps by isolating six novel bacterial strains from paddy soils exhibiting the ability to degrade fipronil, thiobencarb, and a mixture of both pesticides. The degradation efficiency of single isolates and their mixture was evaluated, and optimal degradation conditions were determined using a Box–Behnken design. Furthermore, the study aimed to elucidate novel fipronil and thiobencarb degradation pathways mediated by these isolates and their mixtures. Finally, comprehensive bioaugmentation experiments were conducted in paddy soil microcosms with various conditions using selected isolates and their mixtures.

## 2 Materials and methods

### 2.1 Chemicals and culture media

Fipronil and thiobencarb (purity exceeding 98%) were procured from Golsam Co. and Kavosh Kimia Kerman Co., Iran, respectively (further details provided in Supplementary Material Section A, Supplementary Table 1). All solvents used in this study were of analytical grade (99.9% purity). Anhydrous magnesium sulfate (MgSO_4_) and sodium chloride (NaCl) were acquired from BioShop, Canada (reagent grade, 98% purity). Fipronil and thiobencarb stock solutions (1 g/L) were prepared in acetone for soil spiking and in methanol for subsequent chromatographic analysis. All solutions were stored at −20°C.

A mineral salt medium (MSM; Cycoń et al., 2009) was utilized for the enrichment process. This medium was composed of the following components per liter of deionized water: 2 g (NH_4_)_2_SO_4_, 0.2 g MgSO_4_.7H_2_O, 0.01 g CaCl_2_.2H_2_O, 0.001 g FeSO_4_.7H_2_O, 1.5 g Na_2_HPO_4_.12H_2_O, 1.5 g KH_2_PO_4_, and 0.5 g K_2_HPO_4_. A yeast peptone glucose agar (YPGA) medium including 5 g yeast extract, 5 g peptone, 10 g glucose, and 18 g agar per liter of deionized water was prepared and used for growing and purifying bacterial cultures. Media were sterilized and their pH was adjusted to 7.0.

### 2.2 Soil collection

A rice field near Amol City, Mazandaran province, Iran was selected for soil sampling. This area has a documented history of extensive fipronil and thiobencarb pesticide use (further details in Supplementary Material Section B and Supplementary Table 2).

### 2.3 Enrichment and isolation of degrading bacterial strains

Following the previously described enrichment method (Faridy et al., 2024), fipronil- and thiobencarb-degrading bacteria were enriched through sequential cycles in MSM containing either fipronil, thiobencarb or a mixture of both pesticides at 25 and 50 μg/ml. To isolate pure strains capable of degrading the pesticides, a serial dilution method was employed on MSM agar plates containing the respective pesticides (all at 50 μg/ml). This process resulted in the isolation of two fipronil-degrading isolates (FA and FB), three thiobencarb-degrading isolates (TA, TB, TC), and three isolates degrading the fipronil + thiobencarb mixture (MA, MB, MC). All isolates were further purified on YPGA plates and cryopreserved in glycerol stocks at −20°C.

### 2.4 Evaluating the growth and degradation capabilities of the isolates

Erlenmeyer flasks containing MSM supplemented with pesticides (50 μg/ml) were inoculated with single isolates or their mixtures at a concentration of 4 × 10^7^ cells/ml. The treatments included fipronil with single isolates (FA, FB) or their mixture (FM), thiobencarb with single isolates (TA, TB, TC) or their mixture (TM), and the fipronil + thiobencarb mixture with single isolates (MA, MB, MC) or their mixture (MM). These experiments were conducted in triplicates, with further control flasks without any isolate inoculation for comparison. Over a 14-day incubation period under controlled conditions (30°C, 120 rpm, darkness), samples were periodically withdrawn from each flask. These samples were then analyzed using high-performance liquid chromatography (HPLC) to evaluate pesticide degradation and UV–vis spectrophotometry at 600 nm to assess bacterial growth (OD_600_). Finally, the bacterial isolates or their mixtures that demonstrated the highest degradation efficiency and growth were chosen for further investigation.

### 2.5 Characterization and identification of fipronil and thiobencarb-degrading isolates

Following selection, bacterial isolates underwent characterization according to the protocols outlined in Bergey’s manual of systematic bacteriology. Molecular identification involved genomic DNA extraction (Chen and Kuo, 1993). Subsequently, polymerase chain reaction (PCR) was performed to amplify the 16S rRNA gene using universal bacterial primers: fD1 (5’-AGAGTTGATCCTGGCTCAG-3′) and Rp2 (5′-ACGGCTACCTTGTTACGACTT-3′). The PCR cycling conditions involved an initial denaturation step at 94°C for 5 min, followed by 35 cycles of denaturation (94°C for 30 s), annealing (50°C for 40 s), and extension (72°C for 5 min). Following electrophoresis, the amplified DNA fragments were resolved on a 1% agarose gel for visualization. Subsequently, the Sanger method was employed for sequencing the fragments. The obtained sequences were edited using BioEdit v7.0.9 (Anderson et al., 2003). To identify closely related organisms, a two-pronged approach was undertaken. First, the sequences were compared against the nucleotide collection of the National Center for Biotechnology Information’s (NCBI) BLAST database using the BLASTN algorithm with the “Sequences from type material” filter selected. Additionally, they were compared to the Ezbiocloud 16S-based ID database.^1^ Obtained sequences were submitted to the GenBank database with accession numbers PP657619-PP657624.

### 2.6 Optimization of fipronil and thiobencarb degradation

The degradation rates of fipronil, thiobencarb, and the fipronil + thiobencarb mixture were investigated across a range of concentrations (25–800 μg/ml) using the selected isolates and their mixtures (4 × 10^7^ cells/ml). The Andrews Equation 1 was employed for this analysis (Faridy et al., 2024):


(1) q=qmaxCC+Ks+C2Ki

Within this formula, “C” represents the concentration of fipronil or thiobencarb (μg/ml), “q” signifies the specific degradation rate (day^−1^), “q_max_” indicates the maximum specific degradation rate (day^−1^), “K_s_” denotes the half-saturation constant (μg/ml), and “K_i_” represents the inhibition constant for fipronil or thiobencarb (μg/ml).

A response surface methodology (RSM) with a Box–Behnken design was utilized to examine the influence of pH (5, 7, and 10), pesticide concentration (25, 50, and 100 μg/ml), and inoculum size (OD_600_ ~ 0.01, 0.05, and 0.1) on fipronil and thiobencarb degradation (Pang et al., 2023; Faridy et al., 2024). A total of 17 unique experiments were conducted within this design, each repeated three times for enhanced reliability and precision (Supplementary Material Section C, Supplementary Table 3). The degradation percentages of fipronil and thiobencarb served as the dependent variable for analysis. Finally, a regression (Equation 2) was generated using R 4.3.1.


(2) $$Y_{i}=b_{0}+\sum b_{i}X_{i}+\sum b_{ij}X_{i}X_{j}+\sum b_{ii}X_{i}^{2}$$

In this equation, “Yi” represents the predicted response, “X_i_” and “X_j_” are the variables, and “b_0_,” “b_i_,” “b_ij_,” and “b_ii_” symbolize the coefficients of the corresponding terms. This approach enabled the creation of response surfaces, as described by Pang et al. (2023).

### 2.7 Soil bioaugmentation tests

Soil bioaugmentation tests for fipronil and thiobencarb were conducted in microcosms containing paddy soils without previous pesticide exposure. 40 g of air-dried, sterilized, or non-sterilized soil were placed in individual microcosms. Filter-sterilized aqueous solutions of fipronil, thiobencarb, or a mixture of both pesticides were spiked into the soil at 15 and 150 μg/g dry weight. Moisture was adjusted to 20% or 100% *v/w* to evaluate the impact of moisture on degradation. One group of microcosms was inoculated with pre-selected bacterial isolates or their mixtures (~8 × 10^8^ cells/g) exhibiting biodegradation potential for the target pesticides. The other group remained uninoculated, serving as a control. In total, 120 microcosms were prepared with each treatment replicated three times (n = 3). During a 14-day incubation period at 30°C, subsamples were collected at predetermined intervals for HPLC analysis to quantify the dissipation of fipronil and thiobencarb residues. Further details regarding the soil microcosm design can be found in Faridy et al. (2024) and Supplementary Table 6 (Supplementary Material Section D).

To quantify fipronil and thiobencarb degradation kinetics in the microcosms, a mathematical model was employed. This model assumed a first-order exponential decline, a common approach for degradation processes (equations provided by Torabi et al. (2017)). The model parameters, i.e., degradation rate (k) and half-life (t₁/₂), were estimated by fitting the model to the experimental data.

Analysis of variance (ANOVA) followed by a Tukey’s HSD post-hoc test was used to identify significant degradation percentages (R version 4.3.1.)

The effectiveness of isolate or their mixture addition on pesticide degradation was evaluated by calculating the ratio of degradation rates (k) in inoculated versus uninoculated microcosms (Equation 3). This ratio provides a quantitative measure of the enhancement in degradation due to bioaugmentation.


(3) k+−=kinoculatedsoil/kuninoculatedsoil

### 2.8 Analytical methods

An established unbuffered QuEChERS approach (Anastassiades et al., 2003) was employed to extract fipronil, thiobencarb, and their transformation products (TPs) from the samples. HPLC equipped with a UV/VIS detector (Shimadzu, LC9A) was used to quantify fipronil and thiobencarb. Gas chromatography (GC) coupled with mass spectrometry (MS) using an Agilent 6,890 N GC system interfaced with an Agilent 5973N MS detector with an electron ionization source was employed to detect TPs formed during the degradation process.

A comprehensive validation process was conducted to ensure the accuracy, reliability, and robustness of both the extraction and analysis methods (Corley, 2003; SANTE, 2021). For detailed information on the analytical methods, validation protocols, and results, please refer to Supplementary Material Section E and Supplementary Table 7. Additionally, complete method validation procedures and results are available in our previously published work (Torabi et al., 2024).

## 3 Results

### 3.1 The selection of fipronil and thiobencarb-degrading isolates

**Figure 1:**
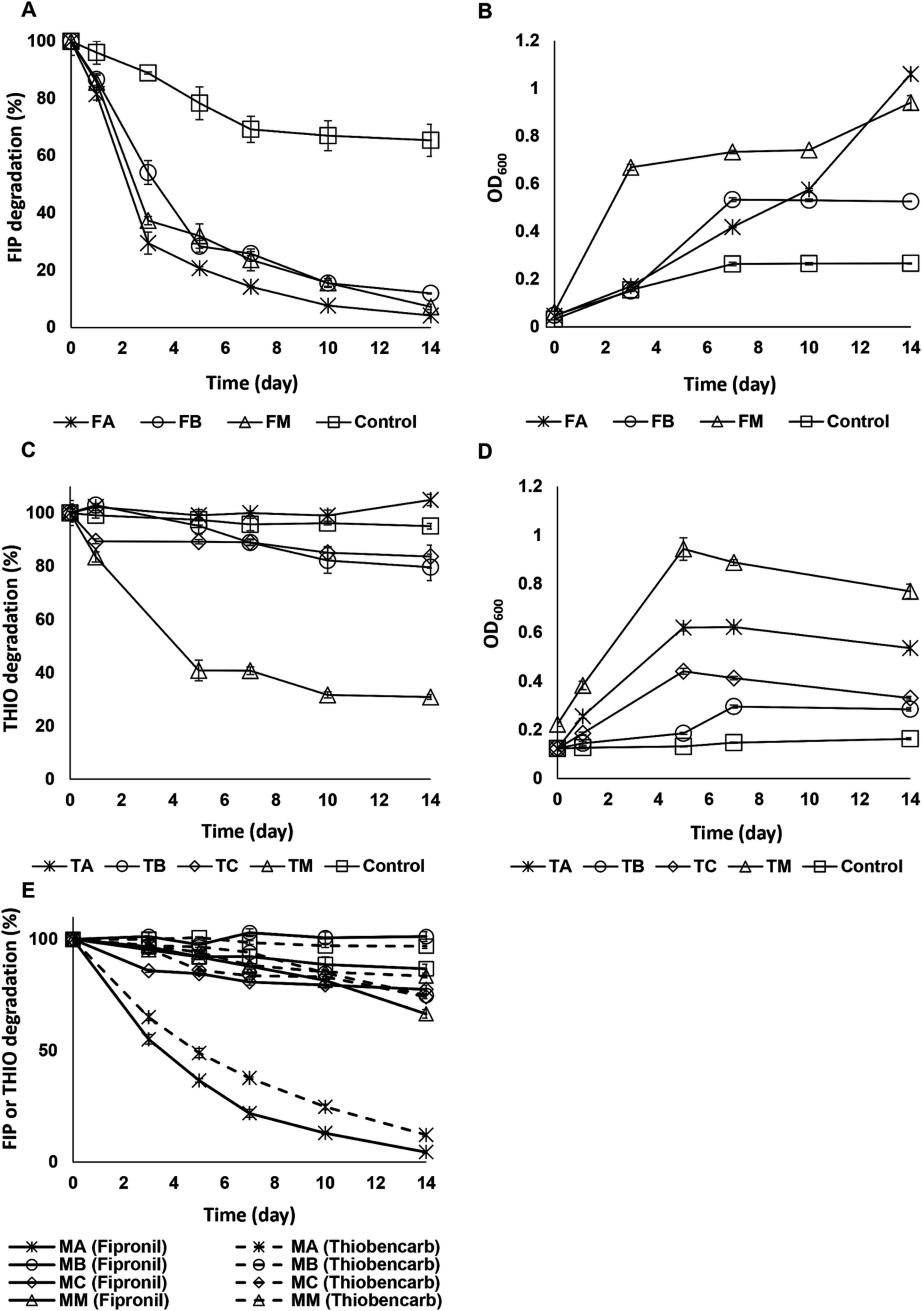
**(A,B)** Fipronil degradation and bacterial growth, respectively in MSM inoculated with FA, FB, and FM, **(C,D)** thiobencarb degradation and bacterial growth, respectively in MSM with TA, TB, TC, and TM, **(E)** fipronil + thiobencarb mixture degradation in MSM inoculated with MA, MB, MC, and MM. In the case of the fipronil + thiobencarb mixture, the initial turbidity of the culture, resulting from adding two pesticide solutions, prevented the measurement of OD_600_. Error bars represent standard deviations (*n* = 3).

Figure 1 illustrates the degradation of fipronil, thiobencarb, and a mixture of fipronil + thiobencarb by isolated strains. When examining fipronil degradation in media, FA, FB, and FM achieved 96, 88, and 93% degradation, respectively. This is substantially higher compared to the control group’s 33% degradation. Additionally, OD_600_ readings showed a 23, 11, and 15-fold increase for FA, FB, and FM, respectively, compared to the control group’s 8-fold increase (Figures 1A,B).

For thiobencarb, TA, TB, and TC exhibited less than 20% degradation within 14 days. However, the mixture TM achieved a 69% degradation rate compared to the control group’s 5%. This was accompanied by a 3-fold increase in culture OD_600_ (Figures 1C,D).

In cultures containing the fipronil + thiobencarb mixture, only MA achieved significant degradation, reaching 96 and 88% for fipronil and thiobencarb, respectively. Other isolates (MB and MC) and their mixture (MM), along with the control group, exhibited a degradation below 33% (Figure 1E).

Following this analysis, isolates FA, FB, and their mixture, FM, were chosen for their ability to degrade fipronil. The consortium TM was selected for thiobencarb degradation and the isolate MA was chosen for its effectiveness in degrading the fipronil + thiobencarb mixture.

### 3.2 Characterization of fipronil and thiobencarb-degrading bacteria

Biochemical characterization results of the selected isolates are presented in Supplementary Table 8. Alignment of the 16S ribosomal RNA sequences revealed a remarkable similarity (greater than 98%) between FA, FB, TA, TB, TC, and MA and specific type strains from GenBank and ezbiocloud 16S-based ID databases (Table 1). FA and FB exhibited a particularly close match to *Enterobacter* sp. and *Brucella* sp. strains. TA, TB, TC, and MA also displayed significant sequence identity with established reference strains of *Stenotrophomonas* sp., *Bordetella* sp. *Citrobacter* sp. and *Pseudomonas* sp., respectively.

**Table 1:** Molecular identification of the selected isolates.

Isolate	Closest relatives in the GenBank/16S-based ID databases (GenBank accession number)	Similarity (%)
FA	*Enterobacter sichuanensis* strain WCHECL1597 (MG832788.1)	99.74
	*Enterobacter chengduensis* strain WCHECl-C4 (KY979142.1)	99.57
	*Enterobacter kobei* strain DSM 13645 (CP017181.1)	99.49
FB	*Brucella ciceri* strain Ca-34 16S (NR_115819.1)	99.34
	*Brucella intermedia* strain LMG 3301 (NR_115045.1)	98.72
	*Brucella abortus* strain 544 (NR_114469.1)	98.04
TA	*Stenotrophomonas pavanii* strain LMG 25348 (NR_118008)	99.85
	*Stenotrophomonas maltophilia* strain ATCC 13637 (NR_112030.1)	99.54
	*Stenotrophomonas humi* strain R-32729 (NR_042568.1)	99.54
TB	*Bordetella muralis* strain T6220-3-2b (NR_145920)	99.42
	*Bordetella tumbae* strain T6713-1-3b (NR_145921.1)	99.35
	*Bordetella tumulicola* strain T6517-1-4b (NR_145922.1)	98.63
TC	*Citrobacter amalonaticus* strain CECT 863 (NR_104823.1)	98.50
	*Citrobacter farmeri* strain CDC 2991–81 (NR_024861.1)	98.32
	*Citrobacter telavivensis* strain 6,105 (NR_180890.1)	97.97
MA	*Pseudomonas urethralis* strain BML-PP042 (NR_181197.1)	99.86
	*Pseudomonas plecoglossicida* strain NBRC 103162 (NR_114226.1)	99.86
	*Pseudomonas monteilii* strain NBRC 103158 (NR_114224.1)	99.58

### 3.3 Optimization of fipronil and thiobencarb degradation conditions

**Figure 2:**
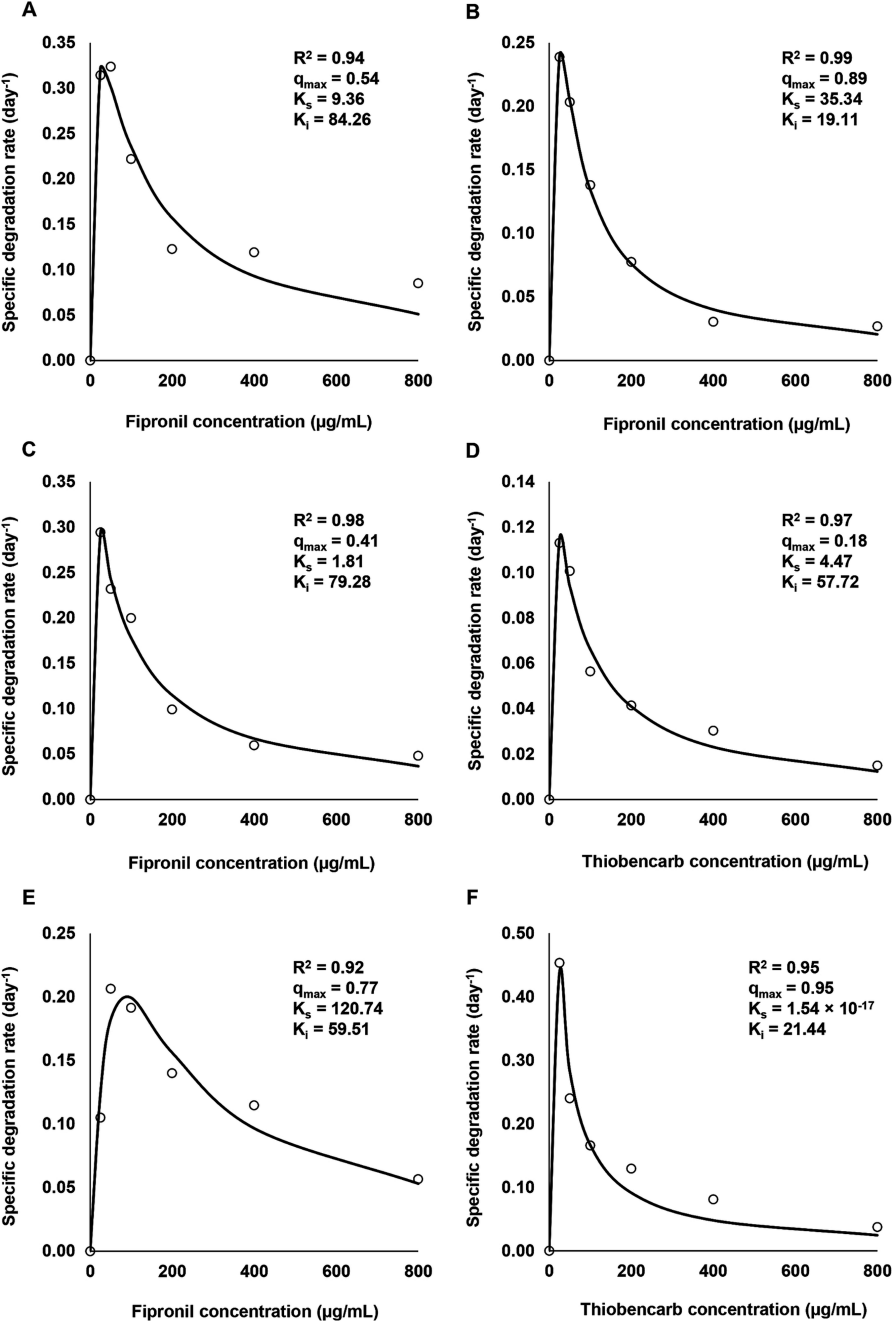
Relationship between initial concentrations of fipronil and thiobencarb and their specific degradation rates by the selected isolates and consortia. **(A–C)**: degradation of fipronil concentrations by FA, FB, and FM, respectively, **(D)**: degradation of thiobencarb concentrations by TM, **(E,F)**: degradation of fipronil and thiobencarb, respectively by MA.

Figure 2 depicts the influence of varying fipronil and thiobencarb concentrations on their degradation kinetic parameters. All isolates and their mixtures exhibited tolerance to elevated pesticide levels (25–800 μg/ml) and successfully degraded both compounds.

For fipronil, isolates FA, FB, and FM displayed the most rapid degradation rates (0.32, 0.24, and 0.29 day^−1^, respectively) at the lowest concentration (25 μg/ml). Isolate MA achieved a peak rate of 0.20 day^−1^ at 100 μg/ml (Figures 2A–C,E). However, higher fipronil concentrations exhibited an inhibitory effect, with the lowest degradation rates observed at 800 μg/ml for all isolates and their mixtures (0.05, 0.02, 0.04, and 0.05 day^−1^ for FA, FB, FM, and MA, respectively; Figures 2A–C,E).

A similar trend emerged for thiobencarb degradation. Isolates TM and MA exhibited the highest rates (0.11 and 0.44 day^−1^, respectively) at 25 μg/ml (Figures 2D,F). Conversely, both isolates displayed evident inhibition at 800 μg/ml, with degradation rates dropping to 0.01 and 0.02 day^−1^ for TM and MA, respectively (Figures 2D,F).

A Box-Benken design with 17 experiments was employed in an RSM regression analysis to optimize fipronil and thiobencarb degradation conditions (Supplementary Table 3). Three independent variables were chosen for this investigation: media pH (X₁), pesticide concentration (X₂), and inoculum size (OD₆₀₀) (X₃). Fipronil degradation achieved by FA, FB, MA, and FM varied between 18.6 to 85.9%, 13.1 to 84.8%, 4.2 to 93.2%, and 28.9 to 95.3%, respectively. Similarly, thiobencarb degradation by TM and MA ranged from 14.7 to 74.0% and 10.1 to 98.1%, respectively (Supplementary Table 3). To mathematically represent the degradation data, quadratic polynomial models were effectively applied for fipronil degradation by FA, FB, FM, and MA (Equations 4–7) and for thiobencarb degradation by TM and MA (Equations 8,9, respectively).


(4) $$\begin{array}{l} Y_{i}=-195.7+55.99X_{1}-0.2926X_{2}+1472X_{3}\\ -0.02616X_{1}X_{2}-167.9X_{1}X_{3}-4.435X_{2}X_{3}\\ -3.285X_{1}^{2}+0.006255X_{2}^{2}+3985X_{3}^{2} \end{array}$$

(5) $$\begin{array}{l} Y_{i}=-314.36+84.16X_{1}-0.098X_{2}+1299.36X_{3}\\ -0.02135X_{1}X_{2}-131.79X_{1}X_{3}-5.764835X_{2}X_{3}\\ -5.000X_{1}^{2}+0.0051X_{2}^{2}+3517.475X_{3}^{2} \end{array}$$

(6) $$\begin{array}{l} Y_{i}=-264.6+73.26X_{1}-0.1651X_{2}+1650.36X_{3}\\ -0.03171X_{1}X_{2}-167.4X_{1}X_{3}-5.505X_{2}X_{3}\\ -4.214X_{1}^{2}+0.006301X_{2}^{2}+2532X_{3}^{2} \end{array}$$

(7) $$\begin{array}{l} Y_{i}=-278.8+111.7X_{1}+0.7381X_{2}-1244X_{3}\\ +0.10551X_{1}X_{2}-3.74X_{1}X_{3}+6.471X_{2}X_{3}\\ -8.026X_{1}^{2}-0.002816X_{2}^{2}+7232X_{3}^{2} \end{array}$$

(8) $$\begin{array}{l} Y_{i}=-258+51.89X_{1}+3.066X_{2}-970.3X_{3}\\ -0.04172X_{1}X_{2}-12.17X_{1}X_{3}-8.132X_{2}X_{3}\\ -3.279X_{1}^{2}-0.0186X_{2}^{2}+359.2X_{3}^{2} \end{array}$$

(9) $$\begin{array}{l} Y_{i}=-461.6+129.1X_{1}+0.192X_{2}+1412X_{3}\\ +0.01667X_{1}X_{2}-104.2X_{1}X_{3}-3.561X_{2}X_{3}\\ -8.283X_{1}^{2}-0.0005969X_{2}^{2}+367.4X_{3}^{2} \end{array}$$

ANOVA results for fipronil and thiobencarb degradation are presented in Supplementary Tables 4 and 5, respectively. All models showed a satisfactory prediction of the degradation data according to their high coefficient of determination (R^2^ > 0.9), significant *F* values (*p* < 0.01), and non-significant lack-of-fit (*p* > 0.05).

The influence of inoculum size (OD₆₀₀) on fipronil degradation exhibited statistically significant effects for FA, FB, FM, and MA (*p* < 0.01), whereas no statistically significant impact of pesticide concentration was observed (*p* > 0.05; Supplementary Table 4). Conversely, for thiobencarb degradation by TM and MA, both inoculum size and pesticide concentration exerted statistically significant effects (*p* < 0.01; Supplementary Table 5). Notably, pH also significantly influenced (*p* < 0.01) fipronil and thiobencarb degradation, except for FB and MA, where pH displayed no statistically significant effects on fipronil (*p* = 0.79) and thiobencarb (*p* = 0.16) degradation, respectively (Supplementary Tables 4 and 5).

**Figure 3:**
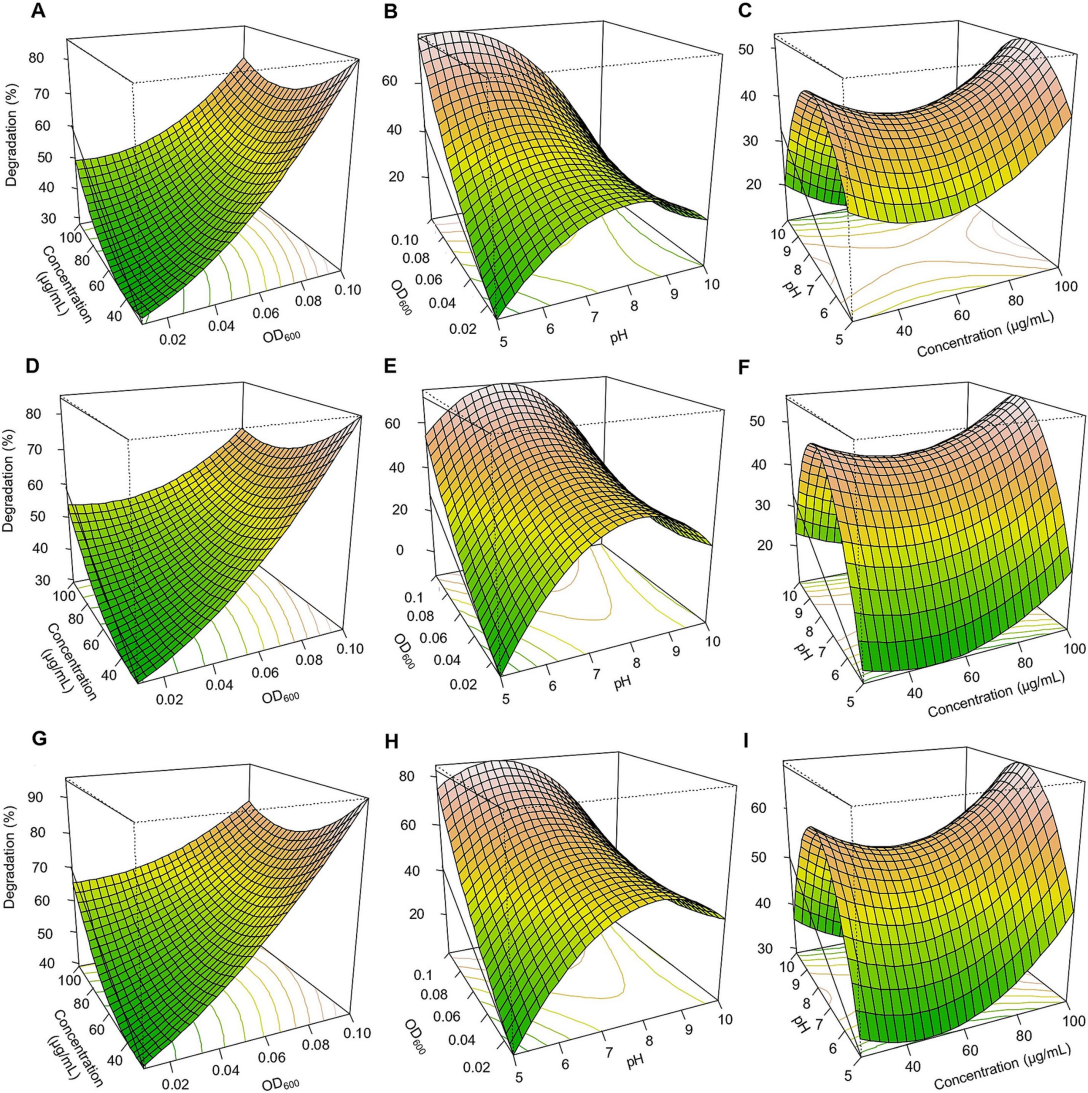
Response surface 3D graphs for fipronil degradation optimization by FA, FB, and FM. **(A,D,G)** effect of pesticide concentration and inoculum size (OD_600_) on fipronil degradation by FA, FB, and FM, respectively, **(B,E,H)** effect of pH and inoculum size (OD_600_) on fipronil degradation by FA, FB, and FM, respectively, **(C,F,I)** effect of pH and pesticide concentration on fipronil degradation by FA, FB, and FM, respectively.

**Figure 4:**
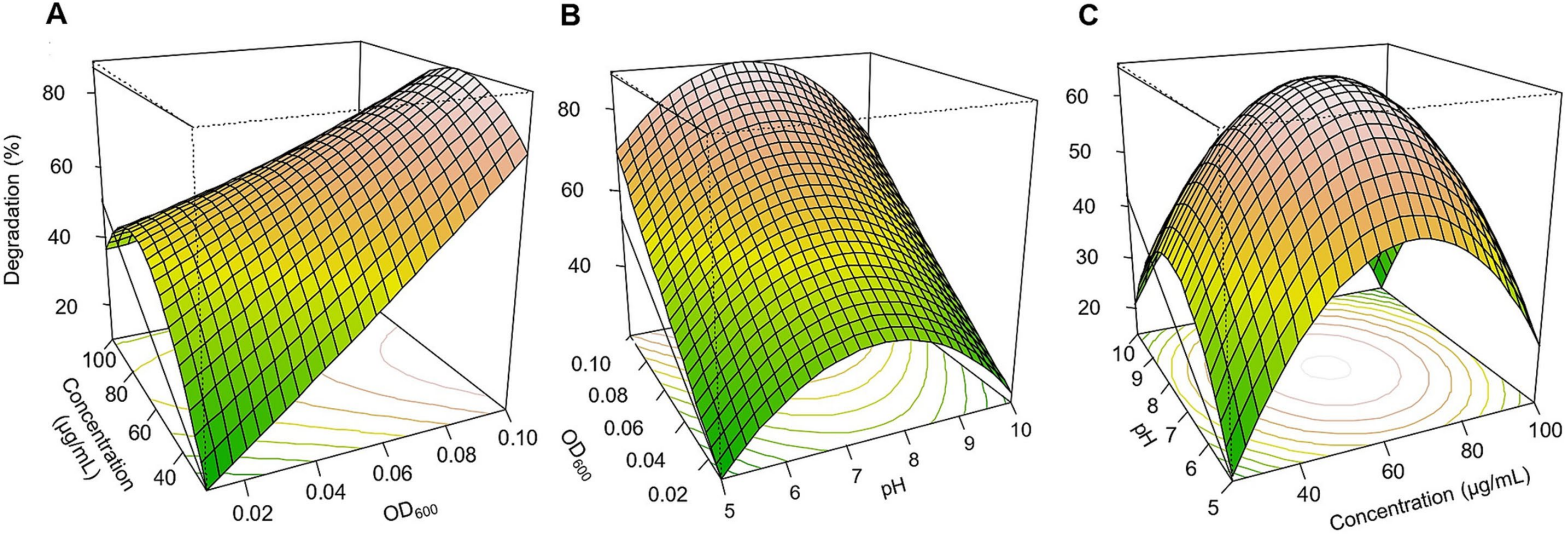
Response surface 3D graphs for thiobencarb degradation optimization by TM. **(A)** Effect of pesticide concentration and inoculum size (OD_600_) on thiobencarb degradation by TM, **(B)** effect of pH and inoculum size (OD_600_) on thiobencarb degradation by TM, **(C)** effect of pH and pesticide concentration on thiobencarb degradation by TM.

**Figure 5:**
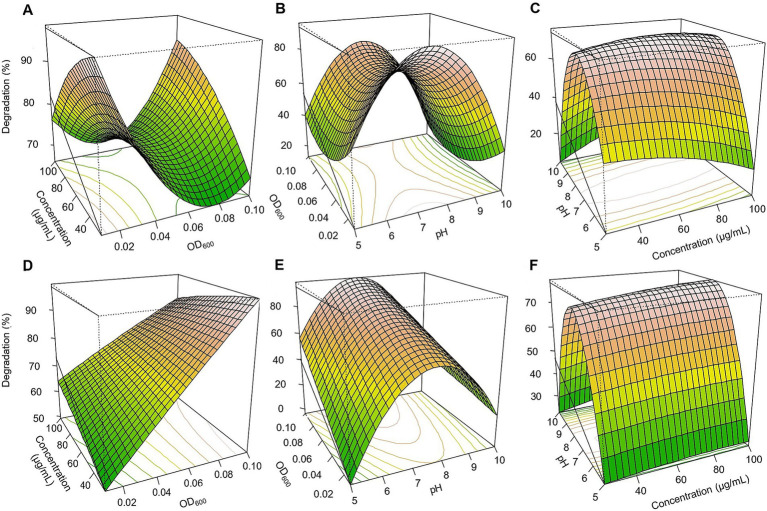
Response surface 3D graphs for fipronil and thiobencarb degradation optimization by MA. **(A,D)** Effect of pesticide concentration and inoculum size (OD_600_) on fipronil and thiobencarb degradation, respectively by MA, **(B,E)** effect of pH and inoculum size (OD_600_) on fipronil and thiobencarb degradation, respectively by MA, **(C,F)** effect of pH and pesticide concentration on fipronil and thiobencarb degradation, respectively by MA.

Based on the three-dimensional response surfaces (Figures 3–5), fipronil optimally degraded at 20–25 μg/ml in a pH of 6–7 with an inoculum size (OD₆₀₀) of 0.01–0.1. These conditions were predicted to achieve approximately 90, 85, 95, and 98% degradation for FA, FB, FM, and MA, respectively (Figures 3, 5A–C). In contrast, for thiobencarb degradation, optimal conditions predicted to achieve approximately 88 and 106% degradation by the TM and MA, respectively, at pH 7, a pesticide concentration of 25–50 μg/ml, and an inoculum size (OD₆₀₀) of 0.1 (Figures 4, 5D–F).

### 3.4 Elucidation of fipronil and thiobencarb degradation pathways

**Figure 6:**
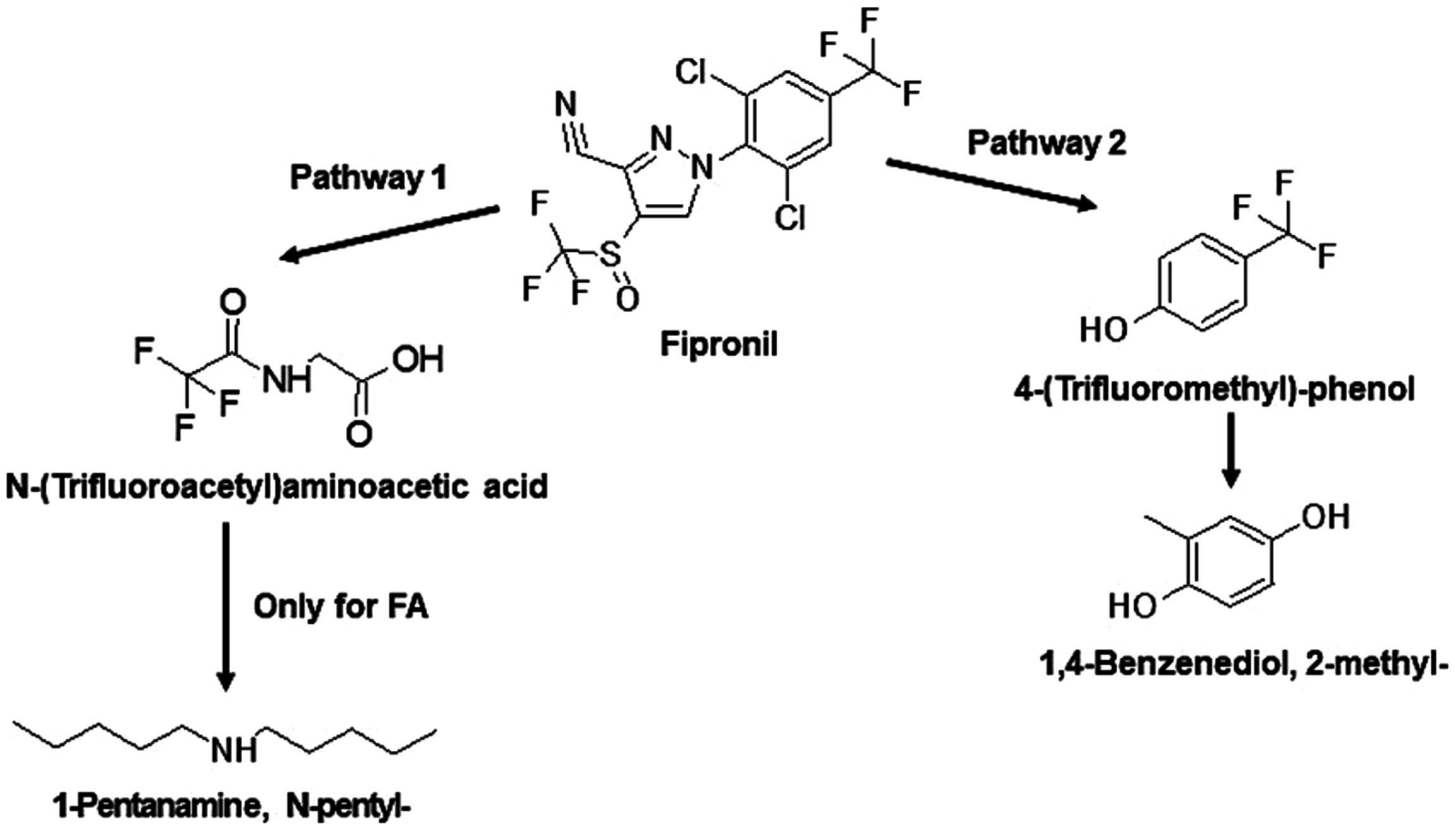
Proposed degradation pathways of fipronil by FA and MA. Fipronil is either transformed to N-(Trifluoroacetyl)aminoacetic acid and 1-Pentanamine, N-pentyl- (pathway 1) or to 4-(Trifluoromethyl)-phenol and 1,4-Benzenediol, 2-methyl- (pathway 2). For MA, transformation to 1-Pentanamine, N-pentyl- was not observed.

**Figure 7:**
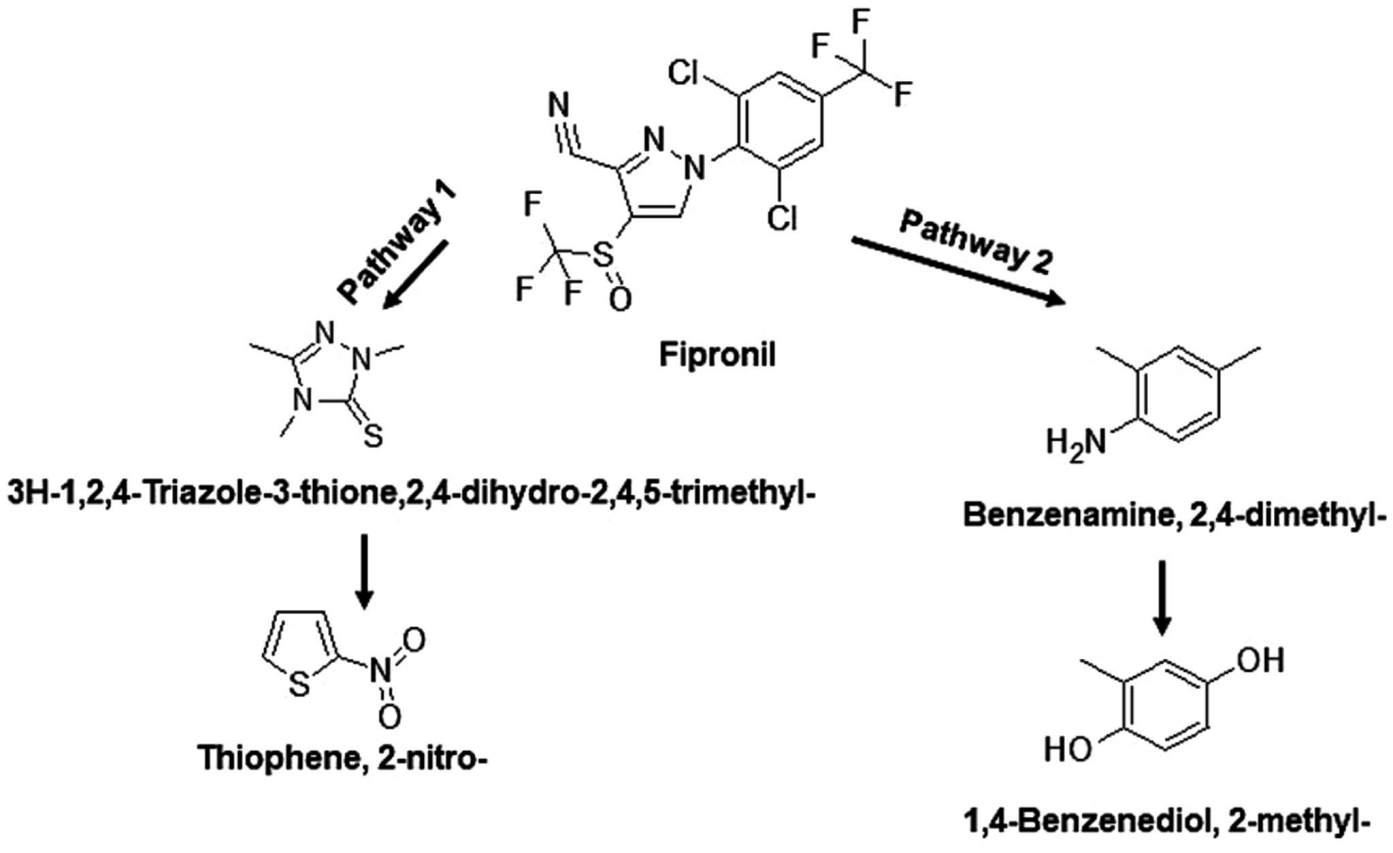
Proposed degradation pathway of fipronil by FB. Fipronil is either transformed to 3H-1,2,4-Triazole-3-thione,2,4-dihydro-2,4,5-trimethyl- and Thiophene, 2-nitro- (pathway 1) or to Benzenamine, 2,4-dimethyl- and 1,4-Benzenediol, 2-methyl- (pathway 2).

**Figure 8:**
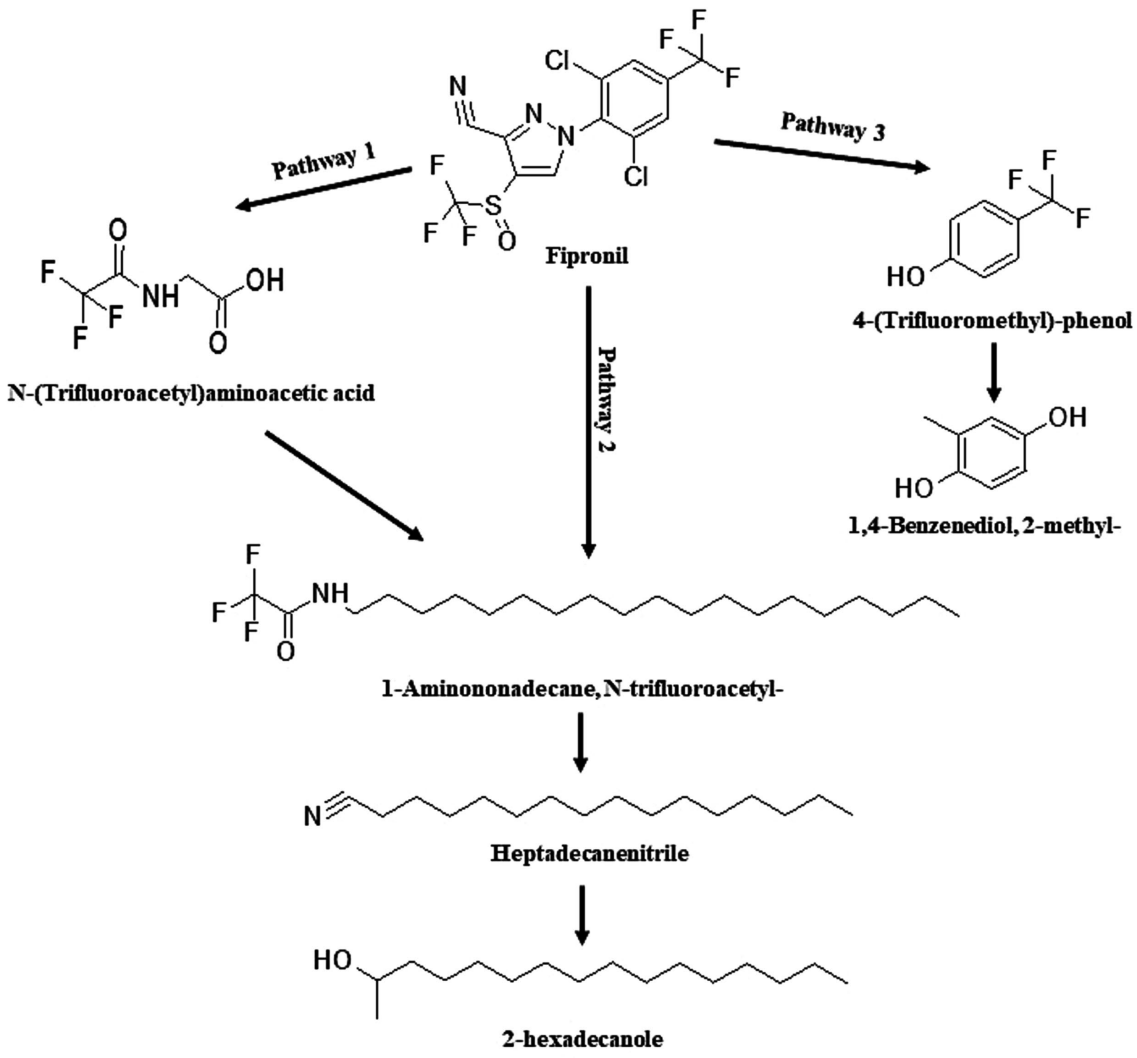
Proposed degradation pathway of fipronil by FM. Fipronil was transformed directly to 1-Aminononadecane, N-trifluoroacetyl-, Heptadecanenitrile, and 2-hexadecanole (pathway 2) or via an intermediate metabolite, N-(Trifluoroacetyl)aminoacetic acid (pathway 1). Alternatively, FM degraded fipronil to 4-(Trifluoromethyl)-phenol and 1,4-Benzenediol, 2-methyl- (pathway 3).

The identified TPs generated during fipronil degradation by FA, FB, FM, and MA are presented in Supplementary Table 9 and Supplementary Figure 1. Based on this metabolic fingerprint, putative degradation pathways for fipronil by each isolate were established (Figures 6–8). Initially, fipronil degradation by FA, FM, and MA appeared to involve a hydrolysis reaction, resulting in the formation of N-(Trifluoroacetyl)aminoacetic acid and 4-(Trifluoromethyl)-phenol. These intermediate TPs were subsequently transformed into 1,4-benzenediol and 1-trifluoroacetoxyhexadecane through potential oxidative or hydrolytic processes (Figures 6, 8). Interestingly, FA exhibited a distinct transformation pathway, resulting in the conversion of N-(Trifluoroacetyl)aminoacetic acid to 1-Pentanamine. In contrast, 1-Pentanamine was not detected in the degradation of fipronil with MA (Figure 6). When FA and FB were combined (FM), the degradation pathway yielded additional products, including 2-hexadecanol and Heptadecanenitrile (Figure 8). FB displayed a distinct pathway, initially converting fipronil to Benzenamine, 2,4-dimethyl- and 3H-1,2,4-Triazole-3-thione,2,4-dihydro-2,4,5-trimethyl- (Figure 7). These intermediary products were further metabolized, primarily through oxidative processes, to generate end products such as Thiophene, 2-nitro- and 1,4-Benzenediol, 2-methyl- (Figure 7).

**Figure 9:**
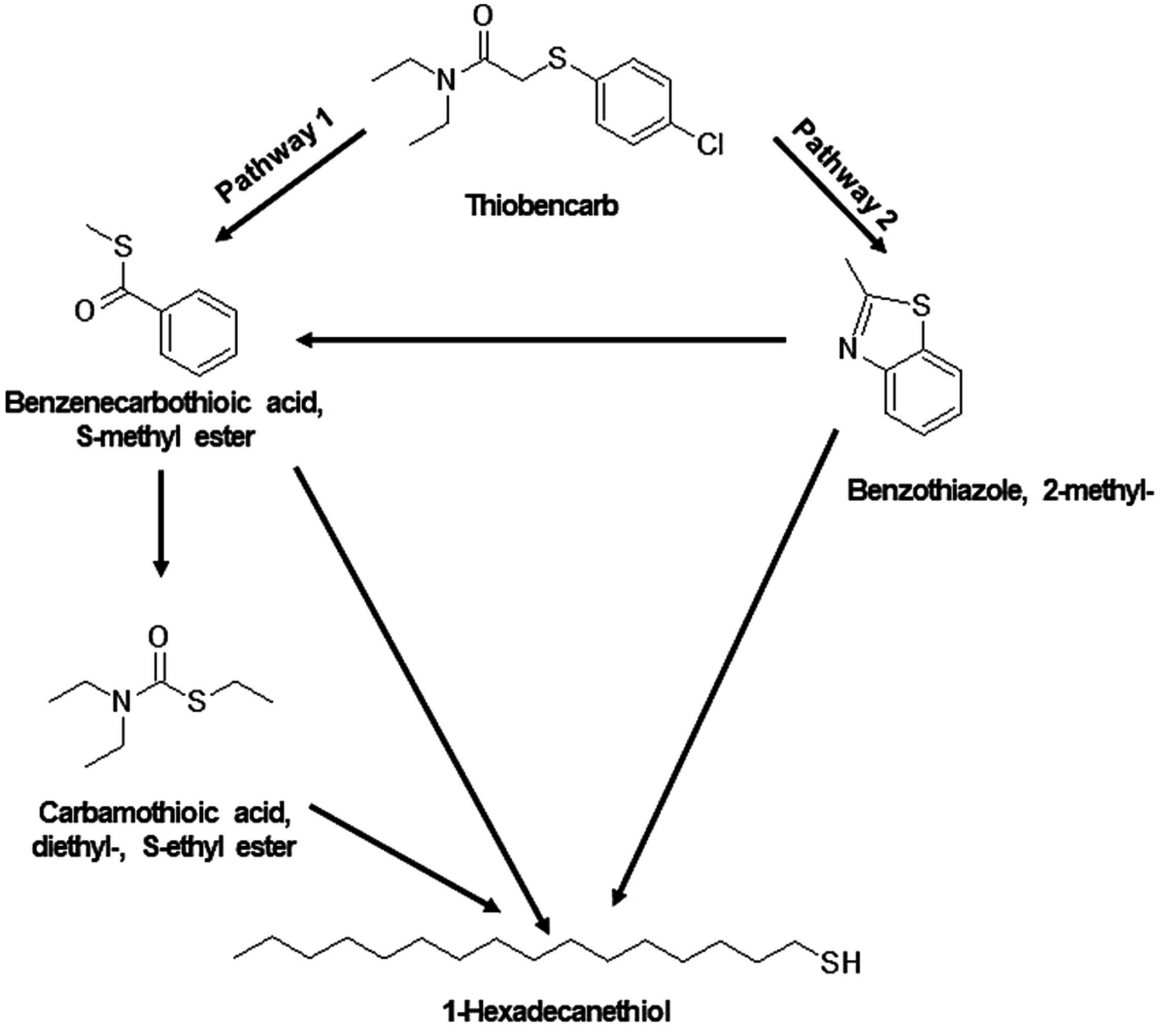
Proposed degradation pathway of thiobencarb by TM and MA. Thiobancarb is either transformed to Benzenecarbothioic acid, S-methyl ester and Carbamothioic acid, diethyl-, S-ethyl ester (pathway 1) or to Benzothiazole, 2-methyl- (pathway 2). Ultimately, both pathways converge to form 1-Hexadecanethiol.

For thiobencarb degradation by TM and MA, comparable TP profiles were observed (Supplementary Table 9 and Supplementary Figure 2). The proposed pathway suggests the transformation of thiobencarb to carbamothioic acid, diethyl-, S-ethyl ester, and benzenecarbothioic acid, S-methyl ester (Figure 9). Additionally, the degradation of THIO by both TM and MA yielded 1-hexadecanethiol and benzothiazole, 2-methyl- as byproducts (Figure 9).

### 3.5 Evaluation of fipronil and thiobencarb degradation in contaminated paddy soil

FA, FB, FM, and MA significantly increased fipronil degradation rates in soils with various sterility and moistures (Tables 2–5). Fipronil t_₁/₂_ in non-inoculated soils ranged from 15 to 129 days (sterile) and 9 to 24 days (non-sterile). Notably, inoculation decreased t_₁/₂_ to 6–22 days and 4–23 days in both sterility conditions, respectively (Tables 2–5). Interestingly, FA, FB, FM, and MA exhibited a more pronounced effect in sterile soils with 20% *v/w* moisture content compared to 100% *v/w* moisture. This resulted in a statistically significant increase (*p* < 0.01) in fipronil degradation and k_(₊/₋)_ values ranging from 7.27 to 14.15 and 6.11 to 9.09 at initial fipronil concentrations of 15 and 150 μg/g, respectively (Tables 2–5). Under high moisture (100% *v/w*), FB and FM exhibited minimal influence (*p* > 0.05) on degradation, while FA remained significantly efficient (*p* < 0.01) with k_(₊/₋)_ values of 2.73 to 3.94 (Tables 2–5). These findings suggest that specific microbial isolates can accelerate fipronil degradation in soil, with efficacy influenced by moisture content.

**Table 2:** Kinetics for fipronil degradation in soil by FA.

Pesticide concentration (μg/g)	Soil Moisture (%)	Soil sterility	Isolate inoculation^*^	D (%) ± SD^**^	t_1/2_ (day)^***^	R^2^^****^	k^*****^ ± SE (day^−1^)	k^******^_(+/−)_ ± SE
15	100	Sterile	+	48.83^f^ ± 1.91	16	0.91	0.044 ± 0.005	0.96 ± 0.11
			−	49.95^ef^ ± 0.96	15	0.97	0.046 ± 0.002	
		Non-sterile	+	83.89^a^ ± 1.51	5	0.99	0.127 ± 0.004	1.63 ± 0.08
			−	66.90^c^ ± 2.09	9	0.96	0.078 ± 0.003	
	20	Sterile	+	59.73^cd^ ± 0.84	11	0.99	0.065 ± 0.001	12.10 ± 0.93
			−	7.23^i^ ± 0.68	129	0.89	0.005 ± 0.000	
		Non-sterile	+	56.11^de^ ± 2.37	12	0.98	0.056 ± 0.002	1.91 ± 0.18
			−	33.33^g^ ± 1.94	24	0.90	0.029 ± 0.003	
150	100	Sterile	+	43.42^f^ ± 1.68	17	0.95	0.040 ± 0.006	3.01 ± 0.23
			−	17.53^h^ ± 0.43	52	0.94	0.013 ± 0.001	
		Non-sterile	+	76.10^b^ ± 3.51	6	0.98	0.119 ± 0.006	1.52 ± 0.08
			−	65.98^c^ ± 1.91	9	0.99	0.078 ± 0.002	
	20	Sterile	+	44.99^f^ ± 2.63	16	0.91	0.043 ± 0.004	6.50 ± 0.67
			−	8.93^i^ ± 0.68	105	0.91	0.007 ± 0.000	
		Non-sterile	+	56.72^de^ ± 0.91	11	0.94	0.063 ± 0.004	2.03 ± 0.23
			−	35.25^g^ ± 3.45	22	0.98	0.031 ± 0.003	

SE: Standard error (*n* = 3). SD: Standard deviation (*n* = 3).

* + and − indicate soils with and without isolate inoculation, respectively.

** Degradation after 14 days. Values with the same lowercase superscript letters in the column do not defer significantly (Tukey’s HSD *post hoc* test, *p* = 0.05).

*** Half-lives of fipronil are calculated based on the first-order exponential decay model (Torabi et al., 2017).

**** The first-order exponential decay model coefficient of determination (Torabi et al., 2017).

***** Fipronil degradation rates based on the first-order exponential decay model (Torabi et al., 2017).

****** The contribution of FA to the degradation rates of fipronil (Equation 3). Standard errors are calculated via the error propagation method.

**Table 3:** Kinetics for fipronil degradation in soil by FB.

Pesticide concentration (μg/g)	Soil Moisture (%)	Soil sterility	Isolate inoculation^*^	D (%) ± SD^**^	t_1/2_ (day)^***^	R^2^^****^	k^*****^ ± SE (day^−1^)	k^******^_(+/−)_ ± SE
15	100	Sterile	+	51.08^c^ ± 1.73	12	0.88	0.059 ± 0.006	1.27 ± 0.13
			−	49.95^c^ ± 0.96	15	0.97	0.046 ± 0.002	
		Non-sterile	+	69.71^b^ ± 4.27	8	0.97	0.090 ± 0.004	1.15 ± 0.07
			−	66.90^b^ ± 2.09	9	0.96	0.078 ± 0.003	
	20	Sterile	+	41.65^cd^ ± 0.62	18	0.87	0.039 ± 0.004	7.27 ± 0.94
			−	7.23^e^ ± 0.68	129	0.89	0.005 ± 0.000	
		Non-sterile	+	84.24^a^ ± 1.22	5	0.99	0.135 ± 0.004	6.67 ± 0.44
			−	33.33^d^ ± 1.94	24	0.90	0.029 ± 0.003	
150	100	Sterile	+	46.46^c^ ± 2.44	17	0.93	0.040 ± 0.003	3.02 ± 0.26
			−	17.53^e^ ± 0.43	52	0.94	0.013 ± 0.001	
		Non-sterile	+	83.92^a^ ± 5.13	4	0.96	0.171 ± 0.011	2.19 ± 0.14
			−	65.98^b^ ± 1.91	9	0.99	0.078 ± 0.002	
	20	Sterile	+	40.02^cd^ ± 7.84	17	0.89	0.040 ± 0.005	6.11 ± 0.90
			−	8.93^e^ ± 0.68	105	0.91	0.007 ± 0.000	
		Non-sterile	+	44.06^cd^ ± 1.29	16	0.97	0.044 ± 0.002	1.41 ± 0.15
			−	35.25^d^ ± 3.45	22	0.98	0.031 ± 0.003	

SE: Standard error (*n* = 3). SD: Standard deviation (*n* = 3).

* + and − indicate soils with and without isolate inoculation, respectively.

** Degradation after 14 days. Values with the same lowercase superscript letters in the column do not defer significantly (Tukey’s HSD *post hoc* test, *p* = 0.05).

*** Half-lives of fipronil are calculated based on the first-order exponential decay model (Torabi et al., 2017).

**** The first-order exponential decay model coefficient of determination (Torabi et al., 2017).

***** Fipronil degradation rates based on the first-order exponential decay model (Torabi et al., 2017).

****** The contribution of FB to the degradation rates of fipronil (Equation 3). Standard errors are calculated via the error propagation method.

**Table 4:** Kinetics for fipronil degradation in soil by FM.

Pesticide concentration (μg/g)	Soil Moisture (%)	Soil sterility	Consortium inoculation^*^	D (%) ± SD^**^	t_1/2_ (day)^***^	R^2^^****^	k^*****^ ± SE (day^−1^)	k^******^_(+/−)_ ± SE
15	100	Sterile	+	65.83^abc^ ± 0.86	9	0.99	0.080 ± 0.002	1.73 ± 0.07
			−	49.95^ef^ ± 0.96	15	0.97	0.046 ± 0.002	
		Non-sterile	+	63.52^bcd^ ± 1.28	9	0.96	0.079 ± 0.004	1.02 ± 0.07
			−	66.90^ab^ ± 2.09	9	0.96	0.078 ± 0.003	
	20	Sterile	+	55.12^de^ ± 1.57	12	0.99	0.060 ± 0.002	11.09 ± 0.90
			−	7.23^j^ ± 0.68	129	0.89	0.005 ± 0.000	
		Non-sterile	+	42.95^fg^ ± 3.92	18	0.94	0.038 ± 0.003	1.31 ± 0.15
			−	33.33^h^ ± 1.94	24	0.90	0.029 ± 0.003	
150	100	Sterile	+	19.78^i^ ± 1.98	22	0.93	0.031 ± 0.002	2.36 ± 0.20
			−	17.53^i^ ± 0.43	52	0.94	0.013 ± 0.001	
		Non-sterile	+	72.21^a^ ± 0.15	7	0.97	0.105 ± 0.005	1.34 ± 0.07
			−	65.98^abc^ ± 1.91	9	0.99	0.078 ± 0.002	
	20	Sterile	+	58.00^cde^ ± 5.66	12	0.96	0.057 ± 0.003	8.64 ± 0.71
			−	8.93^j^ ± 0.68	105	0.91	0.007 ± 0.000	
		Non-sterile	+	70.60^ab^ ± 1.47	8	0.98	0.089 ± 0.003	2.85 ± 0.29
			−	35.25^gh^ ± 3.45	22	0.98	0.031 ± 0.003	

SE: Standard error (*n* = 3). SD: Standard deviation (*n* = 3).

* + and − indicate soils with and without consortium inoculation, respectively.

** Degradation after 14 days. Values with the same lowercase superscript letters in the column do not defer significantly (Tukey’s HSD *post hoc* test, *p* = 0.05).

*** Half-lives of fipronil are calculated based on the first-order exponential decay model (Torabi et al., 2017).

**** The first-order exponential decay model coefficient of determination (Torabi et al., 2017).

***** Fipronil degradation rates based on the first-order exponential decay model (Torabi et al., 2017).

****** The contribution of FM to the degradation rates of fipronil (Equation 3). Standard errors are calculated via the error propagation method.

**Table 5:** Kinetics for fipronil and thiobencarb degradation in soil by MA.

Pesticide	Pesticide concentration (μg/g)	Soil Moisture (%)	Soil sterility	Isolate inoculation^*^	D (%) ± SD^**^	t_1/2_ (day)^***^	R^2^^****^	k^*****^ ± SE (day^−1^)	k^******^_(+/−)_ ± SE
Fipronil	15	100	Sterile	+	80.48^a^ ± 0.27	6	0.99	0.118 ± 0.000	2.56 ± 0.09
				−	49.95^g^ ± 0.96	15	0.97	0.046 ± 0.002	
			Non-sterile	+	75.25^ab^ ± 2.23	7	0.99	0.093 ± 0.004	1.19 ± 0.07
				−	66.90^cd^ ± 2.09	9	0.96	0.078 ± 0.003	
		20	Sterile	+	64.89^de^ ± 0.57	9	0.99	0.076 ± 0.002	14.15 ± 1.09
				−	7.23^j^ ± 0.68	129	0.89	0.005 ± 0.000	
			Non-sterile	+	33.94^h^ ± 1.21	23	0.93	0.030 ± 0.002	1.03 ± 0.12
				−	33.33^h^ ± 1.94	24	0.90	0.029 ± 0.003	
	150	100	Sterile	+	73.53^bc^ ± 2.11	7	0.99	0.103 ± 0.003	7.72 ± 0.41
				−	17.53^i^ ± 0.43	52	0.94	0.013 ± 0.001	
			Non-sterile	+	60.86^de^ ± 1.93	10	0.99	0.071 ± 0.002	0.91 ± 0.04
				−	65.98^d^ ± 1.91	9	0.99	0.078 ± 0.002	
		20	Sterile	+	53.88^fg^ ± 2.02	12	0.97	0.059 ± 0.003	9.01 ± 0.72
				−	8.93^j^ ± 0.68	105	0.91	0.007 ± 0.000	
			Non-sterile	+	58.76^ef^ ± 2.83	12	0.98	0.060 ± 0.002	1.91 ± 0.19
				−	35.25^h^ ± 3.45	22	0.98	0.031 ± 0.003	
Thiobencarb	15	100	Sterile	+	58.10^d^ ± 2.47	10	0.98	0.067 ± 0.003	4.46 ± 1.20
				−	19.04^i^ ± 0.62	46	0.98	0.015 ± 0.004	
			Non-sterile	+	50.25^ef^ ± 0.75	13	0.95	0.052 ± 0.002	1.05 ± 0.05
				−	52.05^e^ ± 0.77	13	0.99	0.052 ± 0.001	
		20	Sterile	+	46.39^f^ ± 0.83	15	0.99	0.046 ± 0.001	5.13 ± 0.42
				−	11.05^j^ ± 0.65	78	0.85	0.009 ± 0.001	
			Non-sterile	+	60.81^d^ ± 0.79	10	0.99	0.068 ± 0.001	1.52 ± 0.06
				−	47.00^f^ ± 2.14	15	0.90	0.045 ± 0.002	
	150	100	Sterile	+	84.24^a^ ± 0.94	5	0.99	0.141 ± 0.004	3.92 ± 0.13
				−	39.48^g^ ± 1.03	19	0.99	0.036 ± 0.001	
			Non-sterile	+	77.27^b^ ± 0.17	4	0.93	0.156 ± 0.014	1.19 ± 0.11
				−	82.51^a^ ± 0.75	5	0.99	0.131 ± 0.002	
		20	Sterile	+	47.30^f^ ± 1.46	16	0.98	0.045 ± 0.001	4.37 ± 0.16
				−	13.10^j^ ± 0.61	68	0.97	0.010 ± 0.000	
			Non-sterile	+	70.09^c^ ± 2.39	7	0.97	0.094 ± 0.005	4.12 ± 0.23
				−	27.53^h^ ± 0.64	30	0.99	0.023 ± 0.001	

SE: Standard error (*n* = 3). SD: Standard deviation (*n* = 3).

* + and − indicate soils with and without isolate inoculation, respectively.

** Degradation after 14 days. Values with the same lowercase superscript letters in the column do not defer significantly (Tukey’s HSD *post hoc* test, *p* = 0.05).

*** Half-lives of fipronil and thiobencarb are calculated based on the first-order exponential decay model (Torabi et al., 2017).

**** The first-order exponential decay model coefficient of determination (Torabi et al., 2017).

***** Fipronil and thiobencarb degradation rates based on the first-order exponential decay model (Torabi et al., 2017).

****** The contribution of MA to the degradation rates of fipronil and thiobencarb (Equation 3). Standard errors are calculated via the error propagation method.

The degradation of thiobencarb by TM and MA followed a similar pattern to fipronil. Adding TM or MA significantly increased (*p* < 0.01) thiobencarb breakdown in both sterility conditions regardless of moisture content (Tables 5, 6). Inoculation with these microbes reduced the t_₁/₂_ of thiobencarb from a range of 19–78 days to 5–58 days in sterile soil and from 5–30 days to 4–17 days in non-sterile soil (Tables 5, 6).

**Table 6:** Kinetics for thiobencarb degradation in soil by TM.

Pesticide concentration (μg/g)	Soil Moisture (%)	Soil sterility	Consortium inoculation^*^	D (%) ± SD^**^	t_1/2_ (day)^***^	R^2^^****^	k^*****^ ± SE (day^−1^)	k^******^_(+/−)_ ± SE
15	100	Sterile	+	25.96^g^ ± 1.55	32	0.98	0.022 ± 0.001	2.46 ± 0.22
			−	11.05^j^ ± 0.65	78	0.85	0.009 ± 0.001	
		Non-sterile	+	52.05^d^ ± 0.77	13	0.95	0.052 ± 0.001	1.07 ± 0.07
			−	48.75^de^ ± 0.85	13	0.99	0.052 ± 0.001	
	20	Sterile	+	52.60^d^ ± 0.84	13	0.99	0.054 ± 0.001	3.61 ± 0.97
			−	19.04^h^ ± 0.62	46	0.98	0.015 ± 0.004	
		Non-sterile	+	47.30^e^ ± 0.40	14	0.90	0.051 ± 0.005	1.06 ± 0.11
			−	47.00^e^ ± 2.14	15	0.90	0.045 ± 0.002	
150	100	Sterile	+	14.99^i^ ± 0.13	58	0.98	0.012 ± 0.002	1.17 ± 0.05
			−	13.10^ij^ ± 0.61	68	0.98	0.010 ± 0.000	
		Non-sterile	+	82.51^a^ ± 0.75	5	0.99	0.133 ± 0.001	1.08 ± 0.08
			−	75.84^b^ ± 0.75	5	0.93	0.131 ± 0.010	
	20	Sterile	+	69.58^c^ ± 1.25	8	0.99	0.082 ± 0.002	2.28 ± 0.06
			−	39.48^f^ ± 1.03	19	0.99	0.036 ± 0.002	
		Non-sterile	+	42.13^f^ ± 1.94	17	0.99	0.040 ± 0.000	1.74 ± 0.05
			−	27.53^g^ ± 0.64	30	0.99	0.023 ± 0.001	

SE: Standard error (*n* = 3). SD: Standard deviation (*n* = 3).

* + and − indicate soils with and without consortium inoculation, respectively.

** Degradation after 14 days. Values with the same lowercase superscript letters in the column do not defer significantly (Tukey’s HSD *post hoc* test, *p* = *0.05)*.

*** Half-lives of thiobencarb are calculated based on the first-order exponential decay model (Torabi et al., 2017).

**** The first-order exponential decay model coefficient of determination (Torabi et al., 2017).

***** Thiobencarb degradation rates based on the first-order exponential decay model (Torabi et al., 2017).

****** The contribution of TM to the degradation rates of thiobencarb (Equation 3). Standard errors are calculated via the error propagation method.

Similar to fipronil, TM was most effective at degrading thiobencarb in sterile soil with low moisture (20% *v/w*) compared to high moisture (100% *v/w*). This was statistically significant (*p* < 0.01) with k_(₊/₋)_ values of 3.61 and 2.28 at initial thiobencarb concentrations of 15 and 150 μg/g, respectively (Table 6).

In contrast, MA seemed more adaptable to moisture variations. It enhanced thiobencarb degradation at both high and low moisture levels in sterile soil, achieving k_(₊/₋)_ values of 5.13 and 4.46 at 100 and 20% *v/w* moisture, respectively, with an initial thiobencarb concentration of 15 μg/g (Table 5). This adaptability was further supported by MA’s effectiveness at both moisture levels in sterile soil with a higher initial concentration (150 μg/g) of thiobencarb (k_(₊/₋)_ values of 4.37 and 3.92) and even in non-sterile soil with low moisture (k_(₊/₋)_ value of 4.12; Table 5). These findings suggest MA might be more versatile in degrading thiobencarb under different moisture conditions compared to TM.

## 4 Discussion

### 4.1 Characterization of fipronil and thiobencarb-degrading isolates

Two isolates, designated FA and FB, and their mixture (FM), exhibited fipronil degradation capabilities. Subsequent analysis identified these isolates as *Enterobacter* sp. and *Brucella* sp., respectively, demonstrating high degrees of similarity (>99%) to species like *E. ludwigii* and *E. cloaceae* according to the GenBank database. *Enterobacter* is a well-established genus of facultatively anaerobic, Gram-negative bacteria belonging to the Enterobacteriaceae family. Numerous studies have documented the ability of *E. ludwigii* and *E. cloaceae* to degrade various pesticides, including pyrethroids (Ramya and Vasudevan, 2020), organophosphates, organochlorines (Abraham et al., 2014; Zhao et al., 2014), chlorimuron-ethyl (Pan et al., 2018), DDT (Suman and Tanuja, 2021), atrazine (Ngigi et al., 2012), and oxamyl (Radwan et al., 2017). Likewise, *Brucella* is a recognized genus of aerobic, Gram-negative bacteria within the Brucellaceae family. Notably, *B. intermedia*, exhibiting >99% similarity to FB in GenBank, has been increasingly reported for its potential in pesticide degradation (Silva et al., 2022; Elango et al., 2023; Shazmin et al., 2023).

A mixture designated TM was chosen for its ability to degrade thiobencarb. This mixture comprised three distinct bacterial strains identified as *Stenotrophomonas* sp., *Bordetella* sp., and *Citrobacter* sp. All three identified bacteria are Gram-negative aerobes. Highly similar species in the GenBank database, such as *S. pavanii*, *S. maltophilia*, *Bordetella muralis*, along *Citrobacter* sp., are documented to possess pesticide degradation capabilities (Goswami and Singh, 2009; Odukkathil and Vasudevan, 2015; Pourbabaee et al., 2018b; Tang et al., 2020; Wu et al., 2022; Zhou et al., 2022; Feng et al., 2023; Saengsanga and Phakratok, 2023). Notably, *S. pavanii* has also been recognized as a rice endophytic bacterium (Feng et al., 2017).

Finally, an isolate designated MA was identified for its ability to degrade the fipronil + thiobencarb mixture. This isolate was subsequently characterized and revealed to be *Pseudomonas* sp. Highly similar species within the *Pseudomonas* genus, such as *P. putida*, are frequently reported for their proficiency in pesticide degradation (Pourbabaee et al., 2018a; Esikova et al., 2023; Chandrasekaran and Paramasivan, 2024).

### 4.2 The efficiency of fipronil and thiobencarb degradation by isolated bacteria

Despite the successful degradation of fipronil and thiobencarb across a range of 25–800 μg/ml by the isolates and their mixtures, elevated pesticide concentrations exhibited a negative correlation with their degradation capacity. This detrimental impact is attributed to the inhibitory effects of high pesticide concentrations on microbial growth, metabolism, and enzyme activity (Pang et al., 2023).

The RSM approach in conjunction with the Box–Behnken design revealed that an inoculum size (OD_600_) ranging from 0.01 to 0.1, coupled with a pH of 7, significantly enhanced the degradation activity of the isolates and their mixtures. Additionally, a pesticide concentration range of 25–50 μg/ml was identified as optimal. RSM has been widely used to evaluate key growth-influencing factors such as pH, pesticide concentration, and inoculum size on the degrading ability of microorganisms (Bhatt et al., 2021b; Pang et al., 2023). For example, Bhatt et al. (2021a, 2021b) adopted RSM to optimize the degradation of fipronil by two *Bacillus* strains.

### 4.3 Delineation of fipronil and thiobencarb degradation pathways by isolated strains

Fipronil transforms into several products, including fipronil sulfide, sulfone, amide, and desulfinyl (Singh et al., 2021). However, our study was conducted under dark conditions. Given that fipronil desulfinyl is predominantly a photoproduct (Singh et al., 2021), its absence in our findings is expected. The intermediate metabolites fipronil sulfone, sulfide, and amide are known to be unstable (Bhatt et al., 2021a). Our analysis at day 14, following >90% parent compound degradation, suggests that these intermediates may have been depleted by this stage, as reported in previous research (Abraham and Gajendiran, 2019; Bhatt et al., 2021a). FA, FB, FM, and MA exhibited a common initial step for fipronil degradation involving the hydrolysis of the C-N bond, followed by a cascade of oxidative and hydrolytic reactions (Figures 6–8). These findings align well with established knowledge, which highlights the critical role of oxidative and hydrolytic processes in fipronil degradation facilitated by microbial isolates (Mandal et al., 2013, 2014). The observed degradation of the parent compounds, coupled with the identification of novel transformation products like N-(Trifluoroacetyl)aminoacetic acid and 4-(Trifluoromethyl)-phenol, suggests that the isolated bacteria are involved in the mineralization of fipronil and the subsequent breakdown of intermediate metabolites, including sulfone, sulfide, and amide, into simpler compounds. These findings align with previous studies (Abraham and Gajendiran, 2019; Bhatt et al., 2021a, 2021b) and support the proposal of a novel fipronil degradation pathway (Figures 6-8).

Shifting the focus to thiobencarb degradation pathways, TM and MA were observed to utilize a process involving the breakdown of the C-S bond. This cleavage resulted in the formation of Benzenecarbothioic acid, S-methyl ester, and subsequent compounds, i.e., Benzothiazole, 2-methyl-, 1-hexadecanethiol, and carbamothioic acid, diethyl-, S-ethyl ester (Figure 9). These findings resonate with the observations reported by Chu et al. (2017), who identified diethylcarbamothioic S-acid as the main thiobencarb TP by *Acidovorax* sp. Furthermore, the detection of TPs like 4-chlorobenzyl mercaptan and S-4-chlorobenzyl ethylthiocarbamate during thiobencarb degradation by *Cupriavidus* sp. *and Pseudomonas* sp. (Duc et al., 2023; Duc, 2023) further corroborates the proposed degradation pathways in our study (Figure 9).

### 4.4 Evaluation of fipronil and thiobencarb degradation in soil bioaugmentation tests

Soil bioaugmentation assays employing the selected isolates and their mixtures were conducted under various conditions encompassing pesticide concentration, soil moisture content, and sterility. These tests revealed that across all evaluated conditions, the isolates and their mixtures remarkably improved the degradation of fipronil and thiobencarb compared to control soils without bacterial inoculation (Tables 2–5).

The isolates and their mixtures showed the most pronounced degradation efficiencies in sterile soils. This phenomenon can be attributed to the absence of indigenous microbial communities, allowing the introduced bacteria to act as the primary drivers of pesticide degradation. However, even in soils with pre-existing microbial populations, the isolates and their mixtures showed efficacy, suggesting the potential for synergistic interactions with the indigenous soil microbiome (Bhatt et al., 2021b; Pang et al., 2023).

Optimal degradation of both fipronil and thiobencarb in soil was observed for all isolates and their mixtures at a moisture content of 20% *v/w* (Tables 2–6). This finding aligns with the primarily aerobic nature of the dominant bacteria within the selected isolates and their mixtures. Notably, only FA and MA exhibited efficient degradation under anoxic conditions prevalent in soils with 100% *v/w* moisture content (Tables 2, 5). This observation can be explained by the facultatively anaerobic nature of most *Enterobacter* and *Pseudomonas* species, allowing them to maintain metabolic activity under oxygen-limited conditions (Ji et al., 2016; Kampers et al., 2021).

## 5 Conclusion

This investigation successfully isolated six bacterial strains from paddy soils exhibiting the ability to degrade fipronil and thiobencarb. Fipronil was degraded with single strains of *Enterobacter* sp. and *Brucella* sp., as well as their combined mixture. A separate mixture, comprised of *Stenotrophomonas* sp., *Bordetella* sp., and *Citrobacter* sp., degraded thiobencarb. Finally, *Pseudomonas* sp. degraded a mixture of fipronil + thiobencarb.

All isolated bacteria demonstrated remarkable efficiency, degrading over 70% of fipronil and thiobencarb within a 14-day incubation period. Notably, these strains exhibited strong toleration and degradation capabilities even at elevated pesticide concentrations, exceeding 800 μg/ml. The primary degradation means employed by the isolates involved oxidation and hydrolysis, potentially representing novel pathways for fipronil and thiobencarb degradation. Furthermore, all isolates and mixtures displayed significant pesticide degradation efficacy across a variety of soil conditions, encompassing diverse pesticide concentrations, moisture levels, and sterility status. In conclusion, this study presents the first report of six novel bacterial strains with promising potential for bioremediation and bioaugmentation applications in paddy soils contaminated with fipronil and thiobencarb. Their exceptional pesticide degradation capabilities, combined with their adaptability to different soil environments, highlight their value as potential tools for mitigating pesticide pollution within paddy soil ecosystems.

## Data Availability

The datasets presented in this study can be found in online repositories. The names of the repository/repositories and accession number(s) can be found at: https://www.ncbi.nlm.nih.gov/genbank/, PP657619-PP657624.

## Footnotes

^1^https://www.ezbiocloud.net
