# Supplementary material for: Unveiling six novel bacterial strains for fipronil and thiobencarb biodegradation: efficacy, metabolic pathways, and bioaugmentation potential in paddy soil
Source: Front Microbiol. 2024 Oct 22;15:1462912. doi: 10.3389/fmicb.2024.1462912 (PMC11536974; doi:10.3389/fmicb.2024.1462912)
Supplement: Supplementary file 1 [file Data_Sheet_1.docx]

**Supplementary Material**

**Unveiling Six Novel Bacterial Strains for Fipronil and Thiobencarb Biodegradation: Efficacy, Metabolic Pathways, and Bioaugmentation Potential in Paddy Soil**

**Nastaran Faridy^1^, Ehssan Torabi^1*^, Ahmad Ali Pourbabaee^2^, Ebrahim Osdaghi^1^, Khalil Talebi^1^**

^1^ Department of Plant Protection, Faculty of Agriculture, University College of Agriculture and Natural Resources, University of Tehran, Karaj, Iran.

^2^ Department of Soil Science, Faculty of Agriculture, University College of Agriculture and Natural Resources, University of Tehran, Karaj, Iran.

**^*^ Correspondence:**Ehssan Torabi

eh_torabi@ut.ac.ir

**Section A. Physicochemical properties of pesticides used in the study**

Supplementary Table 1 Physicochemical properties of fipronil and thiobencarb

| Properties^*^ | Fipronil | Thiobencarb |
| --- | --- | --- |
| IUPAC name | 5-amino-1-[2,6-dichloro-4-(trifluoromethyl)phenyl]-4-(trifluoromethylsulfinyl)pyrazole-3-carbonitrile | S-4-chlorobenzyl diethyl(thiocarbamate) |
| pK_a_ | Not applicable | Not applicable |
| Molecular mass | 437.1 | 257.78 |
| Solubility in water (mg/L) | 3.78 | 28 |
| Vapor Pressure (mPa) | 0.002 | 2.39 |
| Log K_ow_ | 3.75 | 4.23 |
| Henry's Law constant (Pa m³/mol) | 2.31×10^-4^ | 3.68 × 10^-2^ |
| K_oc_ | 825 to 6863 | 309-5000 |
| Hydrolysis | Stable to hydrolysis at pH 5.5 and pH 7, but has a hydrolysis half-life of 28 days at pH 9 | Hydrolysis half-life >1 year (25 °C, pH 4,7, and 9). |

**^*^** Values from PPDB <http://sitem.herts.ac.uk/aeru/ppdb/>

**Section B. Collection of soil samples**

Soil samples for the bacterial isolation process and degradation tests were collected during 2020 and 2021 from a paddy field located in the city of Amol, Mazandaran province, Iran (Longitude: 52°27'54.58''E, Latitude: 36°228'45.14''N, Height: 30 m).

From the field, 4 samples were collected diagonally from the upper soil layer (0 – 30 cm), and a final amount of 10 kg was selected, transferred to the laboratory, and kept at 4 °C. Samples were mixed, homogenized, and sieved (2 mm mesh) for analysis of their physicochemical properties. Physical and chemical properties including texture, bulk density (BD), organic carbon (OC), pH (in water), and cation exchange capacity (CEC) were analyzed according to ISO standard procedures (http://www.iso.org). Soil macro/microelements amounts were measured using atomic absorption spectrophotometry (ICP-AES, ICAP6500, Thermo Fisher Scientific) (Babcsányi et al. 2014). Soil pH was assayed from a soil/distilled water ratio of 1:2.5 with a pH meter (Singh et al. 2003). The soil's electrical conductivity (EC) was analyzed based on the Singh et al. (2003) procedure using the saturated soil extract. Soil field capacity (FC) was measured based on the method of Karpouzas et al. (2004) by saturating and draining the soil for 24 h. Total nitrogen (N_tot_) was detected using a Vario MAX CNS elemental analyzer. A summary of soil physicochemical properties is listed in Table S2.

Supplementary Table 2. Soil physicochemical properties

| Soil texture (USDA classification) | Silty Clay loam |
| --- | --- |
| Sand (%) | 16 (4)^*^ |
| Clay (%) | 34(6) |
| Silt (%) | 50 (3) |
| BD (g/cm3) | 0.94 (0.05) |
| pH (in water) | 8.3 (0.6) |
| EC (ds/m) | 1.27 (0.05) |
| OC (%) | 2.91 (0.4) |
| N_tot_ (%) | 0.32 (0.06) |
| P (mg/kg) | 5.53 (0.4) |
| K (mg/kg) | 88 (7) |
| Fe (mg/kg) | 63.7 (8.9) |
| Zn (mg/kg) | 2.34 (0.07) |
| Mn (mg/kg) | 8.22 (0.8) |
| Cu (mg/kg) | 2.8 (0.05) |
| Na (me/L) | 1.88 (0.2) |
| Ca (me/L) | 8.2 (0.3) |
| Mg (me/L) | 3.4 (0.04) |
| CEC (%) | 18.1 (2.4) |
| FC (%) | 78 (6) |

**^a:^** Means of triplicated with standard deviations in parentheses; **BD**: bulk density; **OC**: organic carbon; **N_tot_**: toral nitrogen; **EC**: electrical conductivity; **CEC**: cation exchange capacity; **FC**: field capacity

**Section C. Box-Behnken experimental design**

Supplementary Table 3 Box-Behnken experimental design and the response of the dependent variable for fipronil and thiobencarb degradation with the selected isolates and consortia

| Experiment No. | Factors | | | Degradation (%) | | | | | |
| --- | --- | --- | --- | --- | --- | --- | --- | --- | --- |
|  | X_1_ | X_2_ | X_3_ | Fipronil (FA) | Fipronil (FB) | Fipronil (FM) | Thiobencarb (TM) | Fipronil (MA) | Thiobencarb (MA) |
| 1 | 7 | 50 | 0.05 | 33.2 | 49.6 | 51.4 | 61.6 | 73.2 | 77.2 |
| 2 | 5 | 25 | 0.05 | 31.5 | 13.1 | 32.3 | 15.5 | 20.8 | 10.1 |
| 3 | 7 | 50 | 0.05 | 38.5 | 41.6 | 55.1 | 61.6 | 74.7 | 78.1 |
| 4 | 7 | 25 | 0.1 | 85.9 | 84.8 | 95.3 | 74.1 | 69.5 | 98.1 |
| 5 | 10 | 50 | 0.01 | 20.1 | 20.8 | 35.4 | 23.5 | 35.5 | 17.4 |
| 6 | 5 | 100 | 0.05 | 43.1 | 18.9 | 46.0 | 25.0 | 15.5 | 14.5 |
| 7 | 5 | 25 | 0.05 | 31.5 | 13.1 | 32.3 | 15.5 | 49.5 | 34.5 |
| 8 | 7 | 100 | 0.1 | 74.4 | 74.5 | 84.5 | 46.8 | 93.2 | 97.2 |
| 9 | 7 | 50 | 0.05 | 49.0 | 42.7 | 58.4 | 42.2 | 57.1 | 57.6 |
| 10 | 7 | 50 | 0.05 | 42.4 | 51.0 | 56.7 | 61.6 | 67.2 | 63.5 |
| 11 | 10 | 100 | 0.05 | 19.4 | 29.2 | 39.3 | 14.7 | 27.0 | 37.5 |
| 12 | 10 | 25 | 0.05 | 19.8 | 23.7 | 36.7 | 20.8 | 4.2 | 23.4 |
| 13 | 7 | 50 | 0.05 | 39.9 | 27.1 | 43.5 | 61.6 | 71.4 | 76.0 |
| 14 | 10 | 100 | 0.1 | 18.6 | 19.2 | 28.9 | 19.1 | 47.0 | 21.8 |
| 15 | 7 | 100 | 0.01 | 50.4 | 50.0 | 65.2 | 36.8 | 74.8 | 56.0 |
| 16 | 5 | 100 | 0.01 | 23.3 | 17.3 | 30.3 | 20.3 | 26.3 | 13.3 |
| 17 | 7 | 50 | 0.05 | 41.4 | 41.0 | 56.2 | 61.6 | 86.4 | 75.0 |

**X_1_:** Culture pH**, X_2_:** pesticide concentration (µg/mL)**, X_3_:** Inoculum size (OD_600_). Each experiment was conducted in triplicates.

Supplementary Table 4. Analysis of variance for the quadratic fipronil degradation models by FA, FB, FM, and MA

| Source | df | FA | | | | FB | | | | FM | | | | MA | | | |
| --- | --- | --- | --- | --- | --- | --- | --- | --- | --- | --- | --- | --- | --- | --- | --- | --- | --- |
|  |  | SS | MS | F-value | *p*-value | SS | MS | F-value | *p*-value | SS | MS | F-value | *p*-value | SS | MS | F-value | *p*-value |
| Model | 9 | 16505.86 | 1833.98 | 128.20 | < 0.01 | 20038.73 | 2226.53 | 56.41 | < 0.01 | 16728.75 | 1858.77 | 96.33 | < 0.01 | 33274.03 | 3697.12 | 67.34 | < 0.01 |
| $X_{1}$ | 1 | 1939.87 | 1939.87 | 135.63 | < 0.01 | 2.93 | 2.93 | 0.07 | 0.79 | 223.91 | 223.91 | 11.60 | < 0.01 | 543.17 | 543.17 | 9.89 | < 0.01 |
| $X_{2}$ | 1 | 15.85 | 15.85 | 1.11 | 0.30 | 14.38 | 14.38 | 0.36 | 0.55 | 5.09 | 5.09 | 0.26 | 0.61 | 1.23 | 1.23 | 0.02 | 0.88 |
| $X_{3}$ | 1 | 4798.55 | 4798.55 | 335.50 | < 0.01 | 4554.59 | 4554.59 | 115.39 | < 0.01 | 3625.95 | 3625.95 | 187.91 | < 0.01 | 3282.09 | 3282.09 | 59.78 | < 0.01 |
| $X_{1}X_{2}$ | 1 | 334.24 | 334.24 | 23.37 | < 0.01 | 241.83 | 241.83 | 6.13 | 0.02 | 434.16 | 434.16 | 22.50 | < 0.01 | 1880.47 | 1880.47 | 34.25 | < 0.01 |
| $X_{1}X_{3}$ | 1 | 1742.67 | 1742.67 | 121.84 | < 0.01 | 924.38 | 924.38 | 23.42 | < 0.01 | 1641.52 | 1641.52 | 85.07 | < 0.01 | 58.74 | 58.74 | 1.07 | 0.31 |
| $X_{2}X_{3}$ | 1 | 327.58 | 327.58 | 22.90 | < 0.01 | 553.44 | 553.44 | 14.02 | < 0.01 | 504.73 | 504.73 | 26.16 | < 0.01 | 697.35 | 697.35 | 12.70 | < 0.01 |
| $X_{1}^{2}$ | 1 | 4847.78 | 4847.78 | 338.94 | < 0.01 | 11386.20 | 11386.20 | 288.47 | < 0.01 | 8103.84 | 8103.84 | 419.97 | < 0.01 | 25531.95 | 25531.95 | 465.05 | < 0.01 |
| $X_{2}^{2}$ | 1 | 1736.02 | 1736.02 | 121.38 | < 0.01 | 1741.01 | 1741.01 | 44.11 | < 0.01 | 1778.33 | 1778.33 | 92.16 | < 0.01 | 265.19 | 265.19 | 4.83 | 0.03 |
| $X_{3}^{2}$ | 1 | 763.31 | 763.31 | 53.37 | < 0.01 | 619.97 | 619.97 | 15.71 | < 0.01 | 411.42 | 411.42 | 21.32 | < 0.01 | 1013.83 | 1013.83 | 18.47 | < 0.01 |
| Residual | 41 | 586.42 | 14.30 | - | - | 1618.31 | 39.5 | - | - | 791.14 | 19.30 | - | - | 2250.96 | 54.90 | - | - |
| Lack of Fit | 13 | 65.16 | 5.01 | 0.27 | 0.99 | 179.81 | 13.83 | 0.27 | 0.99 | 87.91 | 6.76 | 0.27 | 0.99 | 250.11 | 19.24 | 0.27 | 0.99 |
| Pure error | 28 | 521.26 | 18.62 | - | - | 1438.50 | 51.38 | - | - | 703.23 | 25.12 | - | - | 2000.85 | 71.46 | - | - |
| Total | 50 | 17092.28 | 341.85 | - | - | 21657.04 | 433.14 | - | - | 17520.10 | 350.40 | - | - | 35524.99 | 710.50 | - | - |
| Adjusted R^2^ | | 0.96 | | | | 0.91 | | | | 0.95 | | | | 0.92 | | | |

**X_1_:** Culture pH**, X_2_:** pesticide concentration (µg/mL)**, X_3_:** Inoculum size (OD_600_).

Supplementary Table 5. Analysis of variance for the quadratic thiobencarb degradation models by TM and MA

| Source | df | TM | | | | MA | | | |
| --- | --- | --- | --- | --- | --- | --- | --- | --- | --- |
|  |  | SS | MS | F-value | *p*-value | SS | MS | F-value | *p*-value |
| Model | 9 | 22671.24 | 2519.03 | 95.39 | < 0.01 | 43847.61 | 4871.96 | 70.95 | < 0.01 |
| $X_{1}$ | 1 | 365.99 | 365.99 | 13.86 | < 0.01 | 140.25 | 140.25 | 2.04 | 0.16 |
| $X_{2}$ | 1 | 1693.48 | 1693.48 | 64.13 | < 0.01 | 1064.87 | 1064.87 | 15.51 | < 0.01 |
| $X_{3}$ | 1 | 1675.16 | 1675.16 | 63.43 | < 0.01 | 8505.85 | 8505.85 | 123.87 | < 0.01 |
| $X_{1}X_{2}$ | 1 | 393.18 | 393.18 | 14.89 | < 0.01 | 1.27 | 1.27 | 0.02 | 0.89 |
| $X_{1}X_{3}$ | 1 | 24.82 | 24.82 | 0.94 | 0.34 | 629.24 | 629.24 | 9.16 | < 0.01 |
| $X_{2}X_{3}$ | 1 | 1101.17 | 1101.17 | 41.70 | < 0.01 | 211.13 | 211.13 | 3.07 | 0.09 |
| $X_{1}^{2}$ | 1 | 16733.64 | 16733.64 | 633.64 | < 0.01 | 33232.68 | 33232.68 | 483.97 | < 0.01 |
| $X_{2}^{2}$ | 1 | 620.24 | 620.24 | 23.49 | < 0.01 | 62.30 | 62.30 | 0.91 | 0.35 |
| $X_{3}^{2}$ | 1 | 63.56 | 63.56 | 2.41 | 0.13 | 0.02 | 0.02 | 0.00 | 0.99 |
| Residual | 41 | 1082.75 | 26.41 | - | - | 2815.33 | 68.67 | - | - |
| Lack of Fit | 13 | 120.31 | 9.25 | 0.27 | 0.99 | 312.81 | 24.06 | 0.27 | 0.99 |
| Pure error | 28 | 962.34 | 34.37 | - | - | 2502.52 | 89.38 | - | - |
| Total | 50 | 23753.99 | 475.08 | - | - | 46662.94 | 933.27 | - | - |
| Adjusted R^2^ | | 0.94 | | | | 0.93 | | | |

**X_1_:** Culture pH**, X_2_:** pesticide concentration (µg/mL)**, X_3_:** Inoculum size (OD_600_).

**Section D. Soil degradation tests of fipronil and thiobencarb by the selected isolates and consortia**

Paddy soil with no prior exposure to fipronil or thiobencarb for a minimum of two years was employed to assess the degradation proficiency of the selected isolates and consortia. Microcosms consisted of 100 mL amber glass vials with silicone/natural PTFE caps (Interchim®, France). A 0.2 mm syringe filter mounted on a syringe tip was stuck through the vial cap to allow soil-atmosphere exchanges in the vials while limiting water loss and avoiding contamination.

Each microcosm contained 40 grams of air-dried soil, which was either sterilized or remained non-sterilized. Aqueous solutions of fipronil, thiobencarb, and a combination of both pesticides were prepared. Stock solutions of each pesticide in Ace were first introduced into deionized water. Following 24 h for Ace evaporation, the aqueous solutions were filter-sterilized. These solutions were then used to spike soil samples at concentrations of 15 and 150 µg/g. Additionally, the soil moisture content was adjusted to 20% and 100% *v/w*.

Subsequently, the microcosms were categorized into two groups. Inocula of the selected isolates and consortia (~8 × 10^8^ cells/g) were introduced into the first group. Conversely, the second group remained uninoculated, serving as a control for reference purposes. A summary of the soil experimental design is presented in Table S4.

A total of 72 microcosms were prepared in triplicate, resulting in 24 experiments. Incubation of the soil microcosms was conducted in darkness at 30 °C for 14 days. Subsamples were collected at predetermined intervals and subjected to HPLC analysis to quantify the decline in fipronil and thiobencarb residues.

Supplementary Table 6 Summary of soil degradation experimental design

| Microcosms | Pesticide | Isolate/consortium | Pesticide concentration in soil (µg/g) | Soil sterility* | Consortia inoculation | Number of replicates | Incubation period (day) |
| --- | --- | --- | --- | --- | --- | --- | --- |
| 1 | Fipronil | FA | 15 | Sterile | Inoculated | 3 | 14 |
| 2 |  |  |  |  | Uninoculated | 3 | 14 |
| 3 |  |  |  | Non-sterile | Inoculated | 3 | 14 |
| 4 |  |  |  |  | Uninoculated | 3 | 14 |
| 5 |  |  | 150 | Sterile | Inoculated | 3 | 14 |
| 6 |  |  |  |  | Uninoculated | 3 | 14 |
| 7 |  |  |  | Non-sterile | Inoculated | 3 | 14 |
| 8 |  |  |  |  | Uninoculated | 3 | 14 |
| 9 |  | FB | 15 | Sterile | Inoculated | 3 | 14 |
| 10 |  |  |  |  | Uninoculated | 3 | 14 |
| 11 |  |  |  | Non-sterile | Inoculated | 3 | 14 |
| 12 |  |  |  |  | Uninoculated | 3 | 14 |
| 13 |  |  | 150 | Sterile | Inoculated | 3 | 14 |
| 14 |  |  |  |  | Uninoculated | 3 | 14 |
| 15 |  |  |  | Non-sterile | Inoculated | 3 | 14 |
| 16 |  |  |  |  | Uninoculated | 3 | 14 |
| 17 |  | FM | 15 | Sterile | Inoculated | 3 | 14 |
| 18 |  |  |  |  | Uninoculated | 3 | 14 |
| 19 |  |  |  | Non-sterile | Inoculated | 3 | 14 |
| 20 |  |  |  |  | Uninoculated | 3 | 14 |
| 21 |  |  | 150 | Sterile | Inoculated | 3 | 14 |
| 22 |  |  |  |  | Uninoculated | 3 | 14 |
| 23 |  |  |  | Non-sterile | Inoculated | 3 | 14 |
| 24 |  |  |  |  | Uninoculated | 3 | 14 |
| 25 | Thiobencarb | TM | 15 | Sterile | Inoculated | 3 | 14 |
| 26 |  |  |  |  | Uninoculated | 3 | 14 |
| 27 |  |  |  | Non-sterile | Inoculated | 3 | 14 |
| 28 |  |  |  |  | Uninoculated | 3 | 14 |
| 29 |  |  | 150 | Sterile | Inoculated | 3 | 14 |
| 30 |  |  |  |  | Uninoculated | 3 | 14 |
| 31 |  |  |  | Non-sterile | Inoculated | 3 | 14 |
| 32 |  |  |  |  | Uninoculated | 3 | 14 |
| 33 | Fipronil + Thiobencarb | MA | 15 | Sterile | Inoculated | 3 | 14 |
| 34 |  |  |  |  | Uninoculated | 3 | 14 |
| 35 |  |  |  | Non-sterile | Inoculated | 3 | 14 |
| 36 |  |  |  |  | Uninoculated | 3 | 14 |
| 37 |  |  | 150 | Sterile | Inoculated | 3 | 14 |
| 38 |  |  |  |  | Uninoculated | 3 | 14 |
| 39 |  |  |  | Non-sterile | Inoculated | 3 | 14 |
| 40 |  |  |  |  | Uninoculated | 3 | 14 |

^*^ Soils were sterilized by autoclaving thrice at 121 °C for 20 min (Berns et al. 2008).

**Section E. Analytical methods and method validation for fipronil and thiobencarb analysis**

**Extraction**

An established method based on the unbuffered QuEChERS approach (Anastassiades et al., 2003) was employed to extract fipronil, thiobencarb, and their transformation products (TPs). 5 g or mL of either soil or MSM culture was extracted with 10 mL of MeCN. Subsequently, salting-out was performed, involving the addition of 4 g MgSO_4_ and 1 g NaCl to each sample. Following thorough mixing and centrifugation (5000 rpm, 5 min), a 2 mL aliquot of the resulting supernatant was subjected to a dispersive solid-phase extraction (d-SPE) cleanup step utilizing 300 mg MgSO_4_ and 50 mg PSA. After vigorous shaking for 1 min and centrifugation (5000 rpm, 4 min), the supernatant was evaporated to dryness under a stream of nitrogen (N_2_) and reconstituted in 250 μL MeOH (fipronil and thiobencarb) or MeCN (TPs) for subsequent analysis.

**Fipronil and thiobencarb quantification**

For fipronil and thiobencarb quantification, (HPLC equipped with a UV/VIS detector (Shimadzu, LC9A) was utilized. Specific wavelengths of 280 nm and 233 nm were selected for fipronil and thiobencarb, respectively. Chromatographic separation was achieved using a C_18_ column (150 × 4.6 mm, 5 μm) maintained at 40 °C. The mobile phase consisted of a 70:30 (*v/v*) mixture of MeCN and water in isocratic elution mode, with a flow rate of 1 mL/min.

**Detection of TPs**

Gas chromatography-mass spectrometry (GC-MS) was employed to detect the TPs. The instrument utilized was an Agilent 6890N GC equipped with an Agilent 5973N MS detector featuring an electron ionization source (electron energy: 70 eV, solvent delay: 60 min, mass range: 30−250 *m/z*) (Agilent Technologies, USA). Separations were performed using an HP-5ms column (30m × 0.25 mm × 0.25 µm) with helium (purity > 99.999%) as the carrier gas. Detector, ion source, MS transmission line, and quadrupole temperatures were set to optimized values of 320, 230, 280, and 150 °C, respectively.

For fipronil TPs, the carrier gas flow was set at 1.5 ml/min, with an injector temperature of 280 °C in splitless mode and an injection volume of 2 µL. The oven temperature program began at 100 °C for 1 min, then increased to 250 °C at a rate of 10 °C/min for 2 min, and finally raised to 280 °C at a rate of 10 °C/min for 5 min. For thiobencarb TPs, the carrier gas flow was set at 1 ml/min, with the injector temperature of 280 °C in split mode (split ratio: 1:10). The oven temperature program started at 50 °C for 30 s, then increased to 190 °C at a rate of 10 °C/min for 1 min, and finally raised to 280 °C at 10 °C/min for 2 min.

**Precision and accuracy assessment**

The precision and accuracy of each extraction method were evaluated by computing the recoveries and relative standard deviations (RSDs) of fipronil and thiobencarb spiked into pesticide-free soils (at two moisture contents of 20% and 100%) or mineral salt media (MSM), as described below (SANTE 2021). For this, 20 g of soil or 10 ml of MSM were spiked with aqueous solutions of fipronil and thiobencarb in triplicate. The spiking concentrations were 0.2, 1, and 2 µg/g or µg/mL.

**Detection limits and linearity assessment**

The slope (m) and root mean square error (RMSE) of the solvent calibration curve (in MeOH) were employed to ascertain the instrumental detection limit (IDL) and IQL, respectively, employing Eqs. 1 and 2 (Corley 2003).

$IDL \left( \mu g/mL \right)=\frac{3\times RMSE}{m}$ (1)

$IQL \left( \mu g/mL \right)=\frac{10\times RMSE}{m}$ (2)

The estimated method detection (EMDL) and quantification limits were measured according to Eqs. 3 and 4, respectively (Singh et al. 2007):

$EMDL \left( \mu g/mL \right)=\frac{IDL\times V\times100}{\%Recovery\times M}$ (3)

$EMQL \left( \mu g/mL \right)=\frac{IQL\times V\times100}{\%Recovery\times M}$ (4)

V and M are variables that indicate the solution volume used for analysis (measured in mL) and the amount of soil used for extraction (measured in g), respectively.

Linearity was assessed through matrix-matched calibration curves established in blank soil/MSM extracts, and encompassing five concentrations of each pesticide ranging from EMQL to 10 EMQL. The results of the method validation procedure are presented in Table S7. Also, complete method validation procedures and results are available in our previously published work (Torabi et al., 2024).

Supplementary Table 7 Method validation results for fipronil and thiobencarb analysis in soil and MSM cultures

| Pesticide | Matrix Type | Spiked concentrations  (µg/g) (µg/mL) | Recovery (%) | RSD (%) | Linearity  (Matrix-matched calibration curve) | | | EMDL  (µg/g) (µg/mL) | EMQL  (µg/g) (µg/mL) | IDL  (µg/mL) | IQL  (µg/mL) |
| --- | --- | --- | --- | --- | --- | --- | --- | --- | --- | --- | --- |
|  |  |  |  |  | Range  (µg/g) (µg/mL) | Curve equation | R^2^ |  |  |  |  |
| Fipronil | Soil (20% v*/w* moisture) | 0.2 | 90.42 | 1.39 | 0.18 - 1.80 | Y = 18340X + 2107 | 0.96 | 0.05 | 0.18 | 1.14 | 3.81 |
|  |  | 1 | 94.33 | 2.18 |  |  |  |  |  |  |  |
|  |  | 2 | 93.68 | 1.69 |  |  |  |  |  |  |  |
|  | Soil (100% *v/w* moisture) | 0.2 | 109.42 | 1.83 | 0.18 - 1.80 | Y = 24171X - 540 | 0.97 | 0.06 | 0.18 |  |  |
|  |  | 1 | 100.33 | 2.25 |  |  |  |  |  |  |  |
|  |  | 2 | 99.55 | 1.61 |  |  |  |  |  |  |  |
|  | MSM culture | 0.2 | 99.45 | 7.63 | 0.08 – 0.8 | Y = 25712X + 14 | 0.99 | 0.02 | 0.08 |  |  |
|  |  | 1 | 91.25 | 9.54 |  |  |  |  |  |  |  |
|  |  | 2 | 98.36 | 4.23 |  |  |  |  |  |  |  |
| Thiobencarb | Soil (20% v*/w* moisture) | 0.2 | 91.42 | 2.41 | 0.14 – 1.40 | Y = 89526X+ 10650 | 0.96 | 0.04 | 0.14 | 0.73 | 2.44 |
|  |  | 1 | 85.54 | 1.75 |  |  |  |  |  |  |  |
|  |  | 2 | 89.47 | 3.21 |  |  |  |  |  |  |  |
|  | Soil (100% *v/w* moisture) | 0.2 | 115.42 | 2.75 | 0.11 – 1.1 | Y = 54911X+ 9402 | 0.90 | 0.03 | 0.11 |  |  |
|  |  | 1 | 106.25 | 3.41 |  |  |  |  |  |  |  |
|  |  | 2 | 112.08 | 1.45 |  |  |  |  |  |  |  |
|  | MSM culture | 0.2 | 98.78 | 4.31 | 0.05 - 0.5 | Y = 89462X+ 7157 | 0.93 | 0.01 | 0.05 |  |  |
|  |  | 1 | 97.65 | 5.24 |  |  |  |  |  |  |  |
|  |  | 2 | 92.25 | 8.78 |  |  |  |  |  |  |  |

**RSD**: relative standard deviation of triplicates; **IDL**: instrumental detection limit; **IQL**: instrumental quantification limit; **EMDL**: estimated method detection limit; **EMQL**: estimated method quantification limit

**Section F. Biochemical and molecular properties of the selected isolates**

Supplementary Table 8 Biochemical characteristics of the selected isolates for fipronil and thiobencarb degradation

| Characteristics | Isolates | | | | | | |
| --- | --- | --- | --- | --- | --- | --- | --- |
|  | FA | FB | FC | TA | TB | TC | MA |
| Gram reaction | - | - | - | - | - | - | - |
| Catalase | + | + | + | + | + | + | + |
| Oxidase | - | + | + | - | + | - | + |
| Oxidative/fermentative (O/F) | -/+ | +/- | +/- | -/- | +/- | NA | +/- |
| Nitrate reduction | + | + | + | + | - | + | + |
| Lactose fermentation | + | - | - | - | - | + | - |

NA: Not applicable (facultative anaerobes).

**Section G: Fipronil** **and thiobencarb transformation products (TPs)**

Supplementary Table 9. Intermediate TPs during fipronil and thiobencarb degradation by the selected isolates and consortia

|  | Compound name | RT (min) | MW (g/mol) | Formula |
| --- | --- | --- | --- | --- |
| Fipronil | 1-Pentanamine, N-pentyl- | 10.02 | 157.30 | C_10_H_23_N |
|  | Benzenamine, 2,4-dimethyl- | 10.26 | 121.18 | C_8_H_11_N |
|  | Heptadecanenitrile | 10.30 | 251.45 | C_17_H_33_N |
|  | N-(Trifluoroacetyl)aminoacetic acid | 11.10 | 171.08 | C_4_H_4_F_3_NO_3_ |
|  | 2-hexadecanole | 13.66 | 242.44 | C_16_H_34_O |
|  | 1-Aminononadecane, N-trifluoroacetyl- | 14.70 | 379.5 | C_21_H_40_F_3_NO |
|  | Thiophene, 2-nitro- | 18.25 | 129.14 | C_4_H_3_NO_2_S |
|  | 3H-1,2,4-Triazole-3-thione,2,4-dihydro-2,4,5-trimethyl- | 19.20 | 143.21 | C_5_H_9_N_3_S |
|  | 1,4-Benzenediol, 2-methyl- | 21.26 | 124.14 | C_7_H_8_O_2_ |
|  | 4-(Trifluoromethyl)-phenol | 25.34 | 1692.11 | C_7_H_5_F_3_O |
| Thiobencarb | Benzothiazole, 2-methyl- | 13.80 | 149.21 | C_8_H_7_NS |
|  | 1-Hexadecanethiol | 18.63 | 258.51 | C_16_H_34_S |
|  | Carbamothioic acid, diethyl-, S-ethyl ester | 20.77 | 161.27 | C_7_H_15_NOS |
|  | Benzenecarbothioic acid, S-methyl ester | 22.103 | 152.21 | C_8_H_8_OS |

**RT**: Retention time, **MW**: molecular weight

Supplementary Figure 1 Mass spectra and chemical structures of TPs identified through fipronil degradation by FA, FB, FM, and MA

 Supplementary Figure 2 Mass spectra and chemical structures of TPs identified through thiobencarb degradation by TM and MA

**References**

Anastassiades, M., Lehotay, S. J., Štajnbaher, D., and Schenck, F. J. (2003). Fast and easy multiresidue method employing acetonitrile extraction/partitioning and “dispersive solid-phase extraction” for the determination of pesticide residues in produce. *J. AOAC Int.* 86, 412-431. doi: 10.1093/jaoac/86.2.412

Babcsányi, I., Imfeld, G., Granet, M., and Chabaux, F. (2014). Copper stable isotopes to trace copper behavior in wetland systems. *Environ. Sci. Technol.* 48, 5520–5529. [doi: 10.1021/es405688v](https://doi.org/10.1021/es405688v)

Berns, A. E., Philipp, H., Narres, H. D., Burauel, P., Vereecken, H., and Tappe, W. (2008). Effect of gamma‐sterilization and autoclaving on soil organic matter structure as studied by solid state NMR, UV and fluorescence spectroscopy. *Eur. J. Soil Sci.* 59, 540-550. [doi: 10.1111/j.1365-2389.2008.01016.x](https://doi.org/10.1111/j.1365-2389.2008.01016.x)

Corley J. (2003). “Best practices in establishing detection and quantification limits for pesticide residues in foods” in Handbook of Residue Analytical Methods for agrochemicals, ed. P.W. Lee, H. Aizawa, A. C. Barefoot, J. J. Murphy (Wiley, Chichester), 59–75

Karpouzas, D. G., Hatziapostolou, P., Papadopoulou‐Mourkidou, E., Giannakou, I. O., and Georgiadou, A. (2004). The enhanced biodegradation of fenamiphos in soils from previously treated sites and the effect of soil fumigants*. Environ. Toxicol. Chem.* 23, 2099-2107. [doi; 10.1897/03-531](https://doi.org/10.1897/03-531)

SANTE (2021). Analytical quality control and method validation procedures for pesticide residues analysis in food and feed SANTE 11312/2021. Retrieved August 5, 2023, from <https://www.accredia.it/en/documento/guidance-sante-11312-2021-analytical-quality-control-and-method-validation-procedures-for-pesticide-residues-analysis-in-food-and-feed/>

Singh, B. K., Walker, A., Morgan, J. A. W., and Wright, D. J. (2003). Role of soil pH in the development of enhanced biodegradation of fenamiphos. *Appl. Environ. Microbiol.* 69, 7035-7043. [doi: 10.1128%2FAEM.69.12.7035-7043.2003](https://doi.org/10.1128%2FAEM.69.12.7035-7043.2003)

Singh, S. B., Foster, G. D., and Khan, S. U. (2007). Determination of thiophanate methyl and carbendazim residues in vegetable samples using microwave-assisted extraction. *J. Chromatogr. A.* 1148, 152-157. <https://doi.org/0.1016/j.chroma.2007.03.019>
